# DNA recognition for virus assembly through multiple sequence-independent interactions with a helix-turn-helix motif

**DOI:** 10.1093/nar/gkv1467

**Published:** 2015-12-15

**Authors:** Sandra J. Greive, Herman K.H. Fung, Maria Chechik, Huw T. Jenkins, Stephen E. Weitzel, Pedro M. Aguiar, Andrew S. Brentnall, Matthieu Glousieau, Grigory V. Gladyshev, Jennifer R. Potts, Alfred A. Antson

**Affiliations:** 1 York Structural Biology Laboratory, Department of Chemistry, University of York, York YO10 5DD, UK; 2 Department of Biology, University of York, York YO10 5DD, UK; 3 Institute of Molecular Biology, University of Oregon, Eugene, Oregon 97403, USA; 4 Department of Chemistry, University of York, York YO10 5DD, UK; 5 Department of Biochemistry, School of Biology, Moscow State University, Moscow 119234, Russian Federation

## Abstract

The helix-turn-helix (HTH) motif features frequently in protein DNA-binding assemblies. Viral *pac* site-targeting small terminase proteins possess an unusual architecture in which the HTH motifs are displayed in a ring, distinct from the classical HTH dimer. Here we investigate how such a circular array of HTH motifs enables specific recognition of the viral genome for initiation of DNA packaging during virus assembly. We found, by surface plasmon resonance and analytical ultracentrifugation, that individual HTH motifs of the *Bacillus* phage SF6 small terminase bind the packaging regions of SF6 and related SPP1 genome weakly, with little local sequence specificity. Nuclear magnetic resonance chemical shift perturbation studies with an arbitrary single-site substrate suggest that the HTH motif contacts DNA similarly to how certain HTH proteins contact DNA non-specifically. Our observations support a model where specificity is generated through conformational selection of an intrinsically bent DNA segment by a ring of HTHs which bind weakly but cooperatively. Such a system would enable viral gene regulation and control of the viral life cycle, with a minimal genome, conferring a major evolutionary advantage for SPP1-like viruses.

## INTRODUCTION

Protein–DNA complexes regulate important biological processes, from genome replication, to DNA packaging and remodelling and gene expression (1,2). Errors in the formation and regulation of these assemblies can lead to dysfunction, compromising the evolutionary fitness of organisms. Proteins bind DNA using different structural motifs, such as zinc fingers, leucine zippers, helix-loop-helix and helix-turn-helix (HTH) motifs (1). Within each class of motifs, there are some that interact with specific DNA sequences or conformations, under certain conditions and others which have little sequence or conformational preference (1,2). For many nucleoprotein complexes, the molecular basis of recognition, assembly and function remains poorly understood. We seek here to elucidate how one such complex forms and functions in the context of virus assembly.

Nucleoprotein complexes play a central role in the production of new virus particles by ensuring that the nascent viral genome is preferentially and specifically packaged within a capsid shell (3–5). For double-stranded DNA (dsDNA) viruses with large genomes such as herpesviruses and many tailed bacteriophages, new copies of the genome are produced predominantly as concatemers (3,5,6). A packaging complex is initiated when the viral small terminase protein recognizes and binds viral DNA among the nucleic acids in the cell. This binding serves to define the boundary between genome copies, and enables specific cleavage of the genome by the large terminase protein. The large terminase subsequently oligomerizes around the newly formed DNA end to assemble the motor while docking to an empty capsid to begin translocation (3). Packaging of downstream genome copies can be initiated in one of two ways: by another *de novo* event as described, or by transfer of the motor bound to the start of the next genome copy to a new capsid once the first copy is loaded. This transfer occurs through as-yet-unknown mechanisms on signalling from a specific DNA sequence (*cos*) or a full capsid (*pac*), or both (3,5,7). *De novo* initiation in many viruses accounts for a third of all virus particles produced (8–10); thus, it represents a significant event in viral DNA packaging.

**Figure 1.**
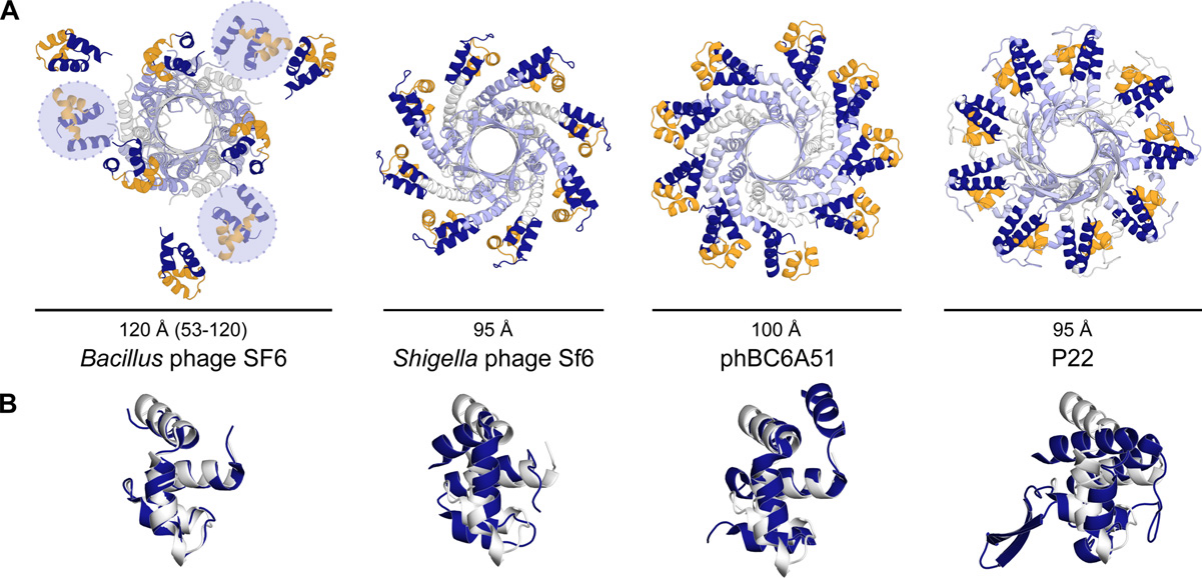
Structural conservation among viral small terminase proteins. (**A**) Cartoon representations of small terminase oligomers, as viewed from the C-terminal end, from *Bacillus* phage SF6, PDB ID: 3ZQQ (14); *Shigella* phage Sf6, PDB ID: 4DYQ (12); *Bacillus* phage phBC6A51, PDB ID: 2AO9; and *Salmonella* phage P22, PDB ID: 3P9A (13). Bars represent the external diameter of the oligomers. Blue circles with dashed lines represent the hypothetical position of unobserved DBDs in SF6 small terminase structure. The oligomerization domain is coloured in alternating white and light blue; DBD-like domain, blue, with helices α2 and α3 of the HTH motif highlighted yellow. (**B**) Secondary structure matching-based alignment of the DBD-like domain of each small terminase with the first 3 α-helices (residues 7–49) of SF6 DBD (white; PDB ID: 4ZC3), oriented in the same direction.

Small terminase proteins of *cos* phages recognize specific sequence elements in the viral genome using HTH motif pairs, much like classical HTH assemblies. For *pac* viruses, however, the mechanism by which small terminase proteins recognize the viral genome is unclear. The majority of these small terminase proteins assemble into ring-like oligomers, consisting of eight to eleven subunits, most with N-terminal HTH motifs arrayed on the outside (Figure 1A) (11–14)—an organization radically different from the classical HTH dimer (1,15). Despite this, deletion or mutation of the HTH motif, for example, in the *Shigella* phage Sf6 and *Bacillus* phage SF6 small terminase (Figure 1B), drastically reduces their affinity towards DNA (12,14). The HTH motifs of *pac* phage small terminase have therefore also been implicated in viral DNA recognition.

HTH motifs are commonly involved in the regulation of DNA replication, repair, transcription and telomere maintenance (15). These motifs generally bind strongly (with nM affinity) to specific DNA sequence repeats as dimers or tetramers (1,15–17). Variations in HTH motifs and their target DNA sequences have led to a fine-tuning of affinity and specificity, giving rise to a range of apparent equilibrium constants from 10^−9^ to 10^−6^ M (18,19). As a result, HTH assemblies are able to take on a wide range of biological roles (1,15).

Previous work on the *Bacillus subtilis* phage SPP1 identified a region on the viral genome that is required for packaging initiation (20). This region, denoted as the *pac* site, contains three sets of direct repeats and spans the promoters and beginning of the small terminase gene. Since HTH motifs commonly bind repeated sequence motifs in the target DNA, the direct repeats identified represent candidate sites for small terminase binding (15,16). Intriguingly, the SPP1 small terminase can be substituted with its homologue from phage SF6 to form viable chimeric virus progeny (21). Despite only moderate conservation in the direct repeats, it appears that the small terminase proteins of the two phages initiate packaging using very similar mechanisms. Here we show that the SF6 DNA-binding domain (DBD) has little specificity towards the identified repeat elements and is, in fact, a generally weak, non-specific binder of both SPP1 and SF6 *pac* DNA. We present detailed analyses of this sequence-independent interaction through multiple complementary biophysical methods. Our observations extend a model where the small terminase protein conformationally selects intrinsically bent DNA (22,23) using circularly arranged but individually weak and non-specific DBDs.

## MATERIALS AND METHODS

### Buffers and reagents

Unless otherwise stated, all surface plasmon resonance (SPR) and analytical ultracentrifugation (AUC) DNA binding analyses were conducted in filtered and degassed 20 mM HEPES, 150 mM KCl, pH 7.0 (binding buffer), with the addition of 0.05% (v/v) Tween 20 (Sigma-Aldrich) for SPR assays. Streptavidin-coated SPR chips were purchased from GE Healthcare Lifesciences (UK) and Xantec bioanalytics GmBH (Dusseldorf, Germany). Modified and unmodified DNA oligonucleotides were purchased from Eurofins Genomics (Ebersberg, Germany) and Integrated DNA Technologies (Leuven, Belgium). DNA and protein concentrations were determined from UV absorbance at 260 and 280 nm respectively, using calculated extinction coefficients based on composition (see Supplementary Table S1 for DNA); 2980 M^−1^ cm^−1^ for SF6 DBD (24–26). The concentration of fluorescently-labelled DNA oligomers was also confirmed at the wavelength for excitation maxima (see Supplementary Table S1). Protein was exchanged into binding buffer by dialysis, ultrafiltration or size-exclusion chromatography. DNA oligomers were dissolved in 10 mM Tris–Cl, pH 8 and diluted into binding buffer. ^15^NH_4_Cl and U-^13^C-glucose for isotope labelling were purchased from Cambridge Isotope Laboratories.

### Cloning, expression and purification of the GP1 DNA-binding domain of Bacillus phage SF6.

DNA sequence encoding the first 68 residues of GP1 was amplified by polymerase chain reaction from the full-length sequence using primers 5′-aggaggggatcatatgaaagaac-3′ and 5′-gtacctcgaggattttcttctcgtttatttccttc-3′ and inserted using the NdeI and XhoI restriction sites into a pET22b expression vector (Novagen) for expression as a fusion protein with a C-terminal 3C protease cleavage site followed by a His-tag. K48A and K26A mutants were created by site-directed mutagenesis using primer pairs 5′-gcagaaaacttacaggcaccagctatccgcgcac-3′, 5′-gtgcgcggatagctggtgcctgtaagttttctgc-3′; and 5′-gacatgaacgctactgcagcggctattgcggca-3′, 5′-tgccgcaatagccgctgcagtagcgttcatgtc-3′, respectively. For structure determination, the wild-type sequence was cloned into a homemade pET28a vector for expression as a cleavable N-terminally His-tagged SUMO-fusion protein, with no linker residues, using the In-Fusion system (Clontech) with primers 5′-gaacagattggtggtatgaaagaacctaaactatctccaaaacag-3′ and 5′-tacctaagcttgtctctattatcagaggattttcttctcgtttatttccttc-3′. Chemically competent *Escherichia* *coli* Stellar cells (Clontech) and BL21(DE3) R3 cells (Structural Genomics Consortium) were used for cloning and protein overproduction, respectively.

For overproduction of unlabelled proteins, cells were grown in LB medium with 30 μg/ml kanamycin. For isotope labelling, cells were grown in M9 minimal medium supplemented with 10 μg/ml riboflavin, niacinamide, pyridoxine hydrochloride, thiamine hydrochloride and trace metals as defined by Studier (27), 30 μg/ml kanamycin and 0.5 g/l ^15^NH_4_Cl and 3 g/l U-^13^C-glucose as required. Fresh medium was inoculated with overnight cultures of transformed cells and incubated at 37°C with shaking until an OD_600_ of 0.8 was reached; then protein expression was induced by addition of IPTG to a final concentration of 1 mM and shaking for 18 h at 16°C.

Cells were harvested by centrifugation and lysed by sonication in 20 mM Tris–Cl, 1 M KCl, 10% (v/v) glycerol, 0.05% (v/v) β-mercaptoethanol, 10 mM imidazole, pH 8.0, with one cOmplete protease inhibitor cocktail tablet (Roche) per litre and 10 μg/ml RNase A. Following centrifugation, the protein was affinity-purified from the soluble fraction using a Ni-charged HiTrap Chelating HP column (GE Healthcare). The column was washed with lysis buffer before proteins were eluted using a 10–300 mM imidazole gradient. Eluate was dialysed overnight at 4°C against 20 mM Tris–Cl, 50 mM KCl, 1 mM DTT, pH 8.0, with 1% (w/w) 3C protease to remove the His-tag. To remove nucleic acid contamination, and since the theoretical pI of the protein is 9.4, cation exchange chromatography was performed, using a Mono S 10/10 column (Amersham). Protein was bound in dialysis buffer without DTT and eluted using a 50–400 mM KCl gradient over 20 CVs. The eluate was concentrated using a 3500 MWCO Amicon Ultra centrifugal filter (Millipore) and passed through a Ni-charged column to sequester uncleaved protein and free tag. Finally the protein was subjected to size-exclusion chromatography on a HiLoad Superdex 75 16/60 column (GE Healthcare) in 20 mM HEPES, 150 mM KCl, pH 7, or 20 mM MES, 150 mM KCl, pH 6.0 for nuclear magnetic resonance (NMR) studies, concentrated and flash-frozen in liquid nitrogen for storage at −80°C. Protein for structure determination was purified as above except that SUMO protease (28) was used to remove the N-terminal tag to produce a native N-terminus.

### Crystallization and structure determination

Crystals were obtained by sitting drop vapour diffusion by mixing 150 nl of protein (20 mg/ml in 25 mM KCl, 10 mM Tris–Cl, pH 7.5) with equal volume of well solution (25% (w/v) PEG 1500, 0.1 M malonate-imidazole-borate pH 5.0) using a Mosquito robot (TTP Labtech). Crystals were transferred into well solution containing an additional 15% (v/v) glycerol before vitrification in liquid nitrogen. X-ray diffraction data were collected at the I04 beamline at Diamond Light Source (Didcot, UK) at 0.9795 Å wavelength on a Pilatus 6M-F detector. The structure was determined by molecular replacement with a 5-residue ideal α-helical fragment placed using Phaser (29). Phases calculated from the placed fragment were improved by density modification with ACORN (30) using normalized structure factors extended to 1.0 Å resolution. The resulting phases were of excellent quality and an essentially complete model could be built with ARP/wARP (31). Subsequent manual rebuilding was performed with Coot (32) and the model refined with REFMAC5 (33) using isotropic ADPs and 4 TLS groups defined by the TLSMD server (34). Structural homologues were identified by secondary structure matching. Subsequent structural alignments were performed in CCP4MG (35,36) and visualized using PyMOL v1.7.6.3 (Schrödinger, LLC).

### Surface plasmon resonance (SPR)

All binding assays were conducted using low-density streptavidin-derivatized (SA) biosensor chips on Biacore instruments (GE Healthcare). Preliminary data were collected on Biacore T100 and T200 instruments at the Bioscience Technology Facility, University of York, the John Innes Centre Surface Plasmon Resonance facility and the Department of Biochemistry Biophysics Facility, University of Cambridge. Final data for analyses were collected using SAD500L biosensor chips (Xantec) on a T200 instrument at the Bioscience Technology Facility, University of York.

Double-referenced equilibrium data were collected, as described by Myszka (37), in serial format with an empty reference flow cell upstream of the ligand-modified flow cell. To reduce errors introduced due to high-nonspecific binding levels, flow cells were modified in an order such that no sample injections would pass over previously modified surfaces. 5′-biotinylated single-stranded (ss) linker oligonucleotides of 20 nt or 30 nt (100 nM, Supplementary Table S1) were immobilized to 400–500 response units (RU) on the chip surface, followed by an injection of complementary ss oligonucleotides (10 μM) or ds oligonucleotides with a 20 nt 3′ ss overhang complementary to the linker to form dsDNA ligands for binding studies. These surfaces were enhanced with a short injection of 1 M KCl, prior to iterative cycles of running buffer or protein diluted in running buffer at 30 μl/min, and a short regeneration pulse of 1 M KCl. Finally, annealed complementary DNA was removed by a 10-s injection of 1 M KCl, 50 mM NaOH, before the next complementary ssDNA strand was annealed. Initial data processing and double referencing were carried out using GE BIAevaluation software, followed by specific data analyses described below.

### ReDCaT surface plasmon resonance screening assay

Modified re-usable DNA capture technology (ReDCaT) assays were conducted as described (38) with the exception that the reference flow channel contained no immobilized DNA, due to the high non-specific binding to ssDNA observed for the HTH domain of the SF6 small terminase. The *pac* sites of SPP1 and SF6 were divided into 30 bp segments that overlap by 5 bp on either end (Supplementary Figure S2 and Table S2). These form the ds region of the complementary oligonucleotide, which has an additional 20 nt 3′ ss overhang in the top strand for annealing with the surface-bound linker. The ds regions were annealed by heating equimolar amounts of each strand to 95°C for 5 min and cooling slowly to room temperature. Each DNA sequence was annealed on-chip to the linker and subjected to protein (20 μM) or buffer injections at least twice. The observed responses at equilibrium were double-referenced against the empty reference flow cell and buffer injection controls. Subsequently, the linker oligonucleotide was regenerated and annealed to the next DNA segment as described above. To control for systematic experimental errors (from flow cell position, experimental time, etc.), binding levels for each sequence were compared to that for a standard sequence repeated at regular intervals during the experimental process. Binding levels were normalized to the amount of DNA oligo annealed for each cycle and the stoichiometry (proteins per DNA) calculated for each sequence as explained below. One-way ANOVA analyses (Microsoft Excel) were used to determine if there were significant differences among the binding levels for all DNA sequences. Post-hoc pairwise comparisons were made using Tukey's method.

### Analysis of non-specific binding by SPR

Equilibrium binding experiments were conducted using 20 and 30 bp dsDNA ligands created by immobilizing 20 and 30 nt 5′-biotinylated oligonucleotides to the SPR chip surface and annealing the complementary oligonucleotide of the same length. A 2-fold dilution series of protein (0.049–1500 μM) was injected in triplicate at 30 μl/min in binding buffer, as defined, over DNA. Equilibrium responses were double-referenced against an empty flow cell and buffer injections across the DNA surfaces. Surfaces were regenerated between protein injections with an injection of 1 M KCl.

To assess non-sequence-specific binding activity, data were analysed under the (Tsodikov-modified) McGhee–von Hippel model (39,40):

(1) \begin{equation*} \frac{v}{L} = K_{\rm a} \left( {1 - nv} \right)\left( {\frac{{1 - nv}}{{1 - (n - 1)v}}} \right)^{n - 1} \left( {\frac{{N - n + 1}}{N}} \right) \end{equation*}

where *v* is the average number of bound proteins per bp DNA, *L* is the free protein concentration, *n* is the integer number of bp the protein occupies (site size), and *N* is the length of the DNA. When the amount of protein injected is in great excess to that of DNA immobilized, *L* approaches the injected protein concentration.

Because protein and DNA have similar refractive index increments (41), the ratio between the SPR equilibrium response, *R*_eq_ and response from DNA immobilization, *R*_L_, is approximately equal to the ratio of masses between protein bound and DNA immobilized. Thus, *v* in Equation (1) can be approximated as follows:

\begin{eqnarray*} R_{\rm eq}&=&R_{\rm L}\cdot{{\rm total\ mass\ of\ protein\ bound}\over{\rm total\ mass\ of\ DNA\ immobilised}} \\ &=&R_{\rm L}\cdot{M_{\rm protein}\cdot{\rm number\ of\ protein\ molecules} \over {M_{\rm DNA}\cdot{\rm number\ of\ DNA\ molecules}}}\\ &=&R_{\rm L}\cdot{M_{\rm protein}\over M_{\rm DNA}}\cdot{\rm average\ number\ of\ protein\ molecules\ per\ DNA\ molecule} \\ &=&R_{\rm L}\cdot{M_{\rm protein}\over M_{\rm DNA}}\cdot{\rm number\ of\ bp\ per\ DNA\ molecule}\cdot{\rm average\ number\ of\ protein\ molecules\ per\ bp\ DNA} \\ &=&R_{\rm L} \bigg ( {M_{\rm protein}\over M_{\rm DNA}} \bigg ) N\nu \end{eqnarray*}

where *M*_protein_ and *M*_DNA_ are the molecular weights of protein and DNA, respectively.

Rearranging we have:

(2) \begin{equation*} v = \frac{{M_{{\rm DNA}} }}{{NM_{{\rm protein}} }} \cdot \frac{{R_{{\rm eq}} }}{{R_{\rm L} }} \end{equation*}

Accordingly, the data were transformed for least squares fitting in R (42), where the residual sum of squares in *v* were solved numerically and minimized by the L-BFGS-B method (43), for each integer value of *n*. Values of *n* and *K*_d_ ( = *K*_a_^−1^) yielding the smallest residual sum of squares and standard error of regression are reported.

### Analytical ultracentrifugation (AUC)

Assembly of the protein–DNA complex was monitored by sedimentation velocity experiments performed on Beckman analytical ultracentrifuges, as previously described (44–47). Analyses of protein assembly onto longer dsDNA oligos (30 bp) were conducted in ultracentrifuge cells containing two-channel epon centerpieces at 20°C and 55 000 rpm for 5 h in an Optima XL-I at the Bioscience Technology Facility, University of York. Smaller species required faster sedimentation speeds to overcome back diffusion and meniscus escape effects (48,49), hence data was collected in ultracentrifuge cells containing two-channel aluminium centerpieces at 20°C and 60 000 rpm for 16 h in a ProteomeLab XL-I at the University of Oregon. Protein and DNA samples were dialysed extensively into binding buffer and protein and DNA concentrations re-determined using UV absorbance values at 280 and 260 nm, respectively, and calculated extinction coefficients (Supplementary Table S1). DNA species (hairpin, 11, 20 and 30 bp) at 5 μM concentration were analysed alone or in the presence of 0.75 or 1.5 mM protein (1 mM for 30mer DNA). Samples were inserted into the sample chamber of the centerpiece while buffer was inserted into the buffer chamber of the centerpiece. Cells were placed in an AN60 titanium rotor and spun at either 55 000 or 60 000 rpm at 20°C for 5 or 16 h respectively. Samples were continuously scanned using the absorbance optics at 495, 649 or 583 nm wavelengths for 6-FAM (hairpin, 20 bp), ATTO 647N (11 bp) and TAMRA (30 bp) labelled dsDNA respectively. Data were analysed using Sedfit (50,51) using continuous c(s) distributions for rapidly associating systems. Generally, fits were considered satisfactory if the root mean squared deviation was less than 0.007, and the residuals were random and less than 10% of the signal. Fit quality and the limitations are discussed further in the supplementary material (SM). Buffer viscosity and density parameters were calculated using Sednterp ((48) Department of Biochemistry, University of New Hampshire). Partial specific volume ($\overline \nu$) for the protein was calculated as described in the SM, while for DNA a measured value of 0.56 was used (52–54). Weight averaged $\overline \nu$ values for different protein–DNA complexes were calculated as described in SM.

### NMR spectroscopy

All spectra were recorded at 25°C in 20 mM MES, 150 mM KCl, pH 6.0, 10% D_2_O, on a Bruker AVANCE II 700 MHz spectrometer with a triple-resonance probe. For spectral assignments using uniformly ^13^C,^15^N-labelled protein, HNCO, HN(CA)CO, CBCANH and CBCA(CO)NH experiments were performed with 50% overall non-uniform sampling (NUS) in the indirect dimensions. To aid verification of backbone assignments and assignment of sidechain resonances, ^13^C-decoupled ^15^N-^1^H TOCSY-HSQC and ^15^N-^1^H NOESY-HSQC experiments were also performed, with mixing times of 60 and 160 ms, respectively, and 40% overall NUS in the indirect dimensions. Data were reconstructed and processed with mddNMR v2.4 (55) and NMRPipe (56), and referenced to 4,4-dimethyl-4-silapentane-1-sulfonic acid (0.1 M) in the sample. Peak lists and assignments are deposited under BMRB entry 25876. For chemical shift perturbation studies DNA and protein were dialysed against the same buffer. Increasing amounts of DNA (7.5 mM) were added to protein (300 μM) to achieve DNA/protein molar ratios of 0.2–19.2 and a series of ^1^H-^15^N HSQC spectra were acquired. Spectral assignments and perturbation data analyses were performed in CcpNmr Analysis v2.4.1 (57). Combined chemical shift differences were given by,

\begin{equation*} \sqrt {\Delta \delta _{\rm H}^2 + \left( {\alpha \cdot \Delta \delta _{\rm N} } \right)^2 } \end{equation*}

where *α* = 0.20 for glycine and *α* = 0.14 for all other residues (58).

## RESULTS

### High-resolution structure of the SF6 small terminase DBD

**Figure 2.**
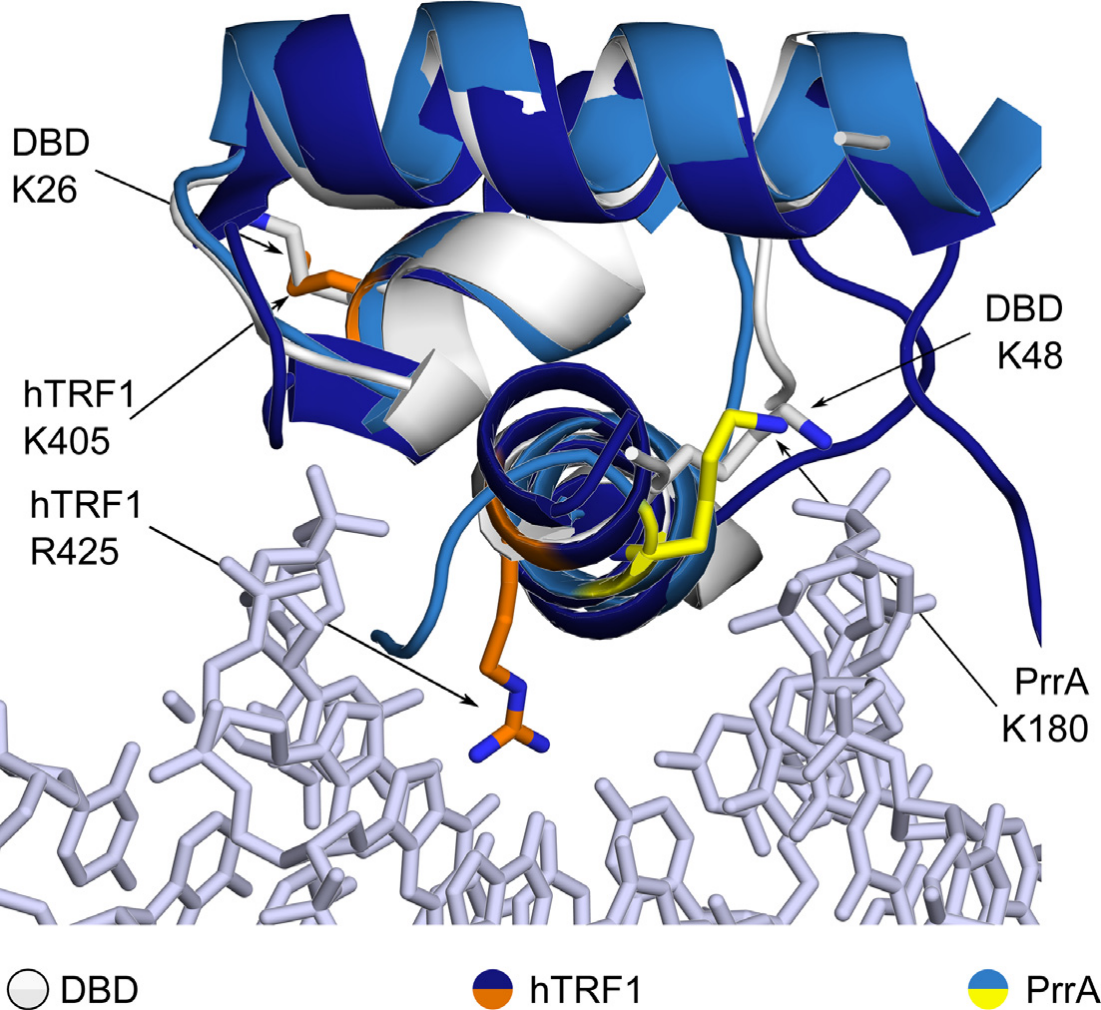
Secondary structure matching-based alignment of SF6 DBD (white, PDB ID: 4ZC3, residues 7–49) with hTRF1 (dark blue, residues 379–430) in complex with DNA (grey; PDB ID: 1W0T) and PrrA (blue, PDB ID: 1UMQ, residues 141–184). Conserved positively charged residues are indicated.

The crystal structure of the small terminase DBD, comprising residues 1–68, was determined to 1.4 Å resolution (PDB ID: 4ZC3; Figures 1B, 2; Supplementary Table S3). In contrast to previous lower-resolution data (14,59), better-defined electron density was observed for terminal residues 6–9 and 60–62. The structure represents a four-helical bundle where helices α2 and α3 form an HTH motif and α4 connects to the central oligomerization domain, as seen in the lower resolution structure of the full length protein (Figure 1A; (14)). The HTH motif is similar to the HTH motifs observed in other small terminase proteins (Figure 1B). The DBDs of the SF6 and SPP1 small terminase share 75% sequence identity, increasing to 79% within the first three helices (Supplementary Figure S1). The two closest structural homologues of SF6 DBD, for which structural information on their interactions with DNA is available, are the human telomerase repeat factor binding protein 1 (hTRF1) (60), and the global response regulator PrrA from *Rhodobacter sphaeroides* (61) (Figure 2 and Supplementary Figure S1; Table S4). Both proteins interact with specific repeat sequences as homodimers, with nanomolar affinities (62,63). Given the structural homology and the presence of box elements within SF6 and SPP1 *pac*, we asked whether SF6 DBD would bind to a specific sequence motif with a similarly high affinity.

### Screening SF6 and SPP1 *pac* site sequences for binding motifs

For many systems, specificity towards the target sequence is achieved through a significant increase in affinity, compensating for the large number of non-specific sites available. So to identify sequences SF6 DBD might target, the SPP1 and SF6 *pac* sites were screened using a modified re-usable DNA capture technology (ReDCaT) SPR assay (38). The two *pac* sites are 80% identical (Supplementary Figure S2), with genomic features such as the promoters, ribosomal binding site and the start codon of small terminase being absolutely conserved. The boxA repeats of SPP1, implicated in small terminase binding, do not occur in SF6, though a different pair of direct repeats can be found in the region. The boxB sequences, which span the nuclease cleavage site for *de novo* packaging initiation and the boxC sequences are 90 and 75% identical, respectively (Supplementary Figure S2).

The *pac* regions were screened in 30-bp segments with 5-bp overlaps on either end (Supplementary Figure S2 and Table S2). Segments from the SPP1 *pac* sequence were designated P#, and segments from SF6, S#. Segments were annealed to a pre-immobilized 20-bp linker to form a 50-bp nicked dsDNA substrate. Because SPR measures changes in mass on the chip surface over time, kinetic parameters and equilibrium binding densities of surface-bound complexes can be determined.

**Figure 3.**
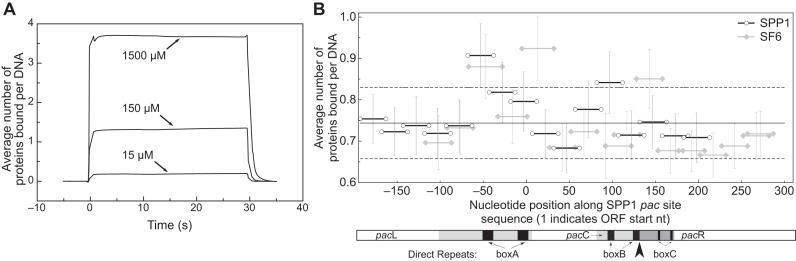
SPR analyses of SF6 DBD binding to *pac* DNA. (**A**) Sensorgrams of DBD binding over time to a nicked 50-bp dsDNA substrate with a 30-bp segment (P6) from a boxA-containing region of SPP1 *pac*. (**B**) Average binding densities (number of DBDs per DNA) to contiguous sequence segments across the aligned SPP1 (black open circles) and SF6 (grey closed diamonds) *pac* in binding buffer containing 150 mM KCl. ‘0’ marks the start of the start codon for small terminase in the SPP1 genome. The grand mean (solid line) ±1 standard deviation (dashed lines) are shown. A diagram of the SPP1 *pac* site is displayed below depicting the left and right ends of the genome (*pac*L and *pac*R, respectively); the direct repeats of boxA, boxB and boxC; and the *pac*C region with the initial large terminase cleavage site indicated by the large arrowhead (88). The region of DNA protected by small terminase (23) is represented by the grey shading in the *pac*L region.

Preliminary analysis with an SPP1 boxA-containing sequence (P6) confirmed that DBD alone could bind DNA, and showed that the binding was superstoichiometric, had rapid kinetics and low affinity, requiring 10^−5^ M concentration for observable binding by SPR, in contrast to the <10^−7^ M required for other HTH monomers binding to cognate sequences (64,65) (Figure 3A). This binding behaviour suggested that DBD did not interact specifically with the boxA sequence (17).

Analysis of all segments showed that DBD bound sequences from both *pac* regions with similar densities, as determined by one-way ANOVA analysis (*F*(31 103) = 1.277, *P* > 0.05, Supplementary Table S5), and with the characteristics of non-specific interactions (Figure 3B). These data suggest that the SF6 small terminase DBD binds *pac* DNA in a non-sequence-specific manner, with rapid kinetics and no apparent preference for any sequence in the region. Under these conditions, it would seem that none of the box repeats, or any sequence motif, were essential for *pac* site recognition by individual DBDs.

#### Effects of salt concentration on binding density

**Figure 4.**
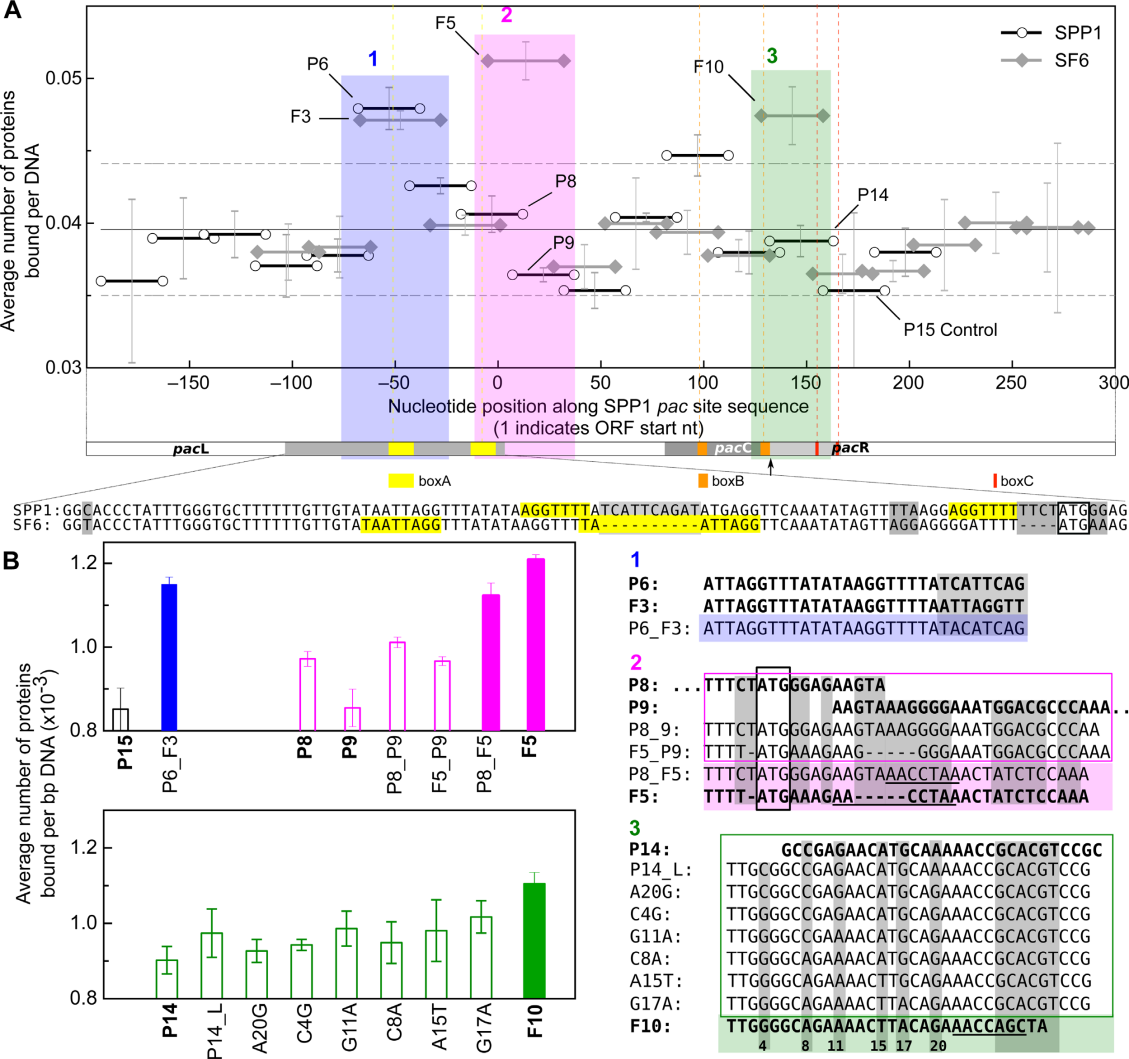
SPR analyses of SF6 DBD binding to *pac* site DNA at high salt concentration. (**A**) Average binding densities (number of DBDs per DNA) to contiguous sequence segments across SPP1 (black open circles) and SF6 (grey closed diamonds) *pac* DNA in binding buffer containing 300 mM KCl. ‘0’ marks the start of the start codon for small terminase in the SPP1 genome. Solid line represents the grand mean, and dashed lines, ±1 standard deviation from the mean. Sequences selected for further analysis are shaded and labelled. Below the graph is a scale diagram of the SPP1 *pac* site depicting the left and right ends of the genome (*pac*L and *pac*R respectively); the direct repeats of boxA (yellow), boxB (orange) and boxC (red); and the pacC region. The relative positions of the repeats are shown in the graph by dashed vertical lines (A, yellow; B, orange; C, red). The specific site cleaved by the large terminase in the initial packaging event is indicated by the arrow (88), while the region of DNA protected by small terminase (23) is represented by the grey shading in the pacL region. Alignment of the SPP1 and SF6 genome sequences in this region is shown below with the box A direct repeats highlighted in yellow, with sequence variations shaded in grey. (**B**) Sequence segments (in genome regions, shaded boxes, 1, blue; 2, magenta; 3, green) with apparent variations in binding density between SPP1 and SF6 were mutated for further analysis. Open bars represent average or below-average relative binding density, while the solid bars represent above-average binding density to the respective sequences that are boxed or shaded accordingly. Wild-type sequences are shown in bold with sequence variation between SPP1 and SF6 shaded in grey. The numbers below region 3 indicate the position relating the bases mutated in each test sequence.

To further examine if the SF6 DBD had sequence preference within the *pac* regions, however weak, we took advantage of the extreme sensitivity of the T200 SPR instrument. The ReDCaT assay was repeated at 300 mM KCl concentration to reduce non-specific interactions (66) so that any preferential binding could be identified (Figure 4A). Binding densities were overall reduced, reflecting the ionic nature of the interactions. The pattern across both *pac* regions was consistent with that observed at 150 mM KCl, though at this higher salt concentration, statistically significant differences could be identified. Binding densities for segments P6 & F3, F5, and F10, were more than one standard deviation above the grand mean (Figure 4A, Regions 1, 2 and 3, respectively), and were statistically different from the other segments, as found by one-way ANOVA (Supplementary Table S5) and post-hoc Tukey's test (*α* = 0.05). The segments did not correlate well with the box repeats, confirming that the repeat sequence motifs had little effect on SF6 DBD binding. Further analyses were performed to identify the preferential binding site.

#### Effects of pac site mutations on binding density

Sequence variations in the highlighted segment regions (Figure 4A) were exploited to identify a motif for preferential DBD binding. Mutant sequences were created and subjected to the ReDCaT assay at 300 mM KCl concentration (Figure 4B). To account for differences in DNA length, binding densities were expressed in number of protein molecules bound per bp DNA. Segments P6 and F3 from Region 1 were identical except for the downstream 8 bp. Mutation of these bases to a random sequence (P6_F3) did not significantly affect binding density. This indicates that any sequence preference would arise from the upstream 22 bp of the segments. In Region 2, SPP1 segments P8 and P9 displayed average binding density, while SF6 segment F5 displayed greater-than-average binding density. A composite segment P8_P9 produced no increased binding relative to the original segments, nor did a hybrid F5_P9. The reverse hybrid P8_F5, however, showed an increase in binding density, approaching that for segment F5. This suggests that the 15 bp at the downstream end of segment F5 may contain a preferred sequence. In Region 3, segment F10 displayed greater-than-average binding density. Sequential point mutations in segment P14 to produce F10 had no significant effect on binding density, suggesting that the preferred sequence occurs instead in the unaligned downstream end of F10.

Analysis of the sequences showing statistically significant greater-than-average binding densities, using the MEME suite (67), identified a potential preferred sequence motif: 5′-[T/G] [T/C] [A/T] GGTT-3′, *p* = 1.17–4.93 × 10^−4^. This motif occurs in the full-length SPP1 and SF6 *pac* sequences, two and three times, respectively (Supplementary Figure S2), and not in scrambled sequences (*p* = 5–70 × 10^−4^, as identified using FIMO (68)). For SPP1, the motifs are in a region known to be protected by small terminase from DNase I digestion (23). This sequence may well help localize the small terminase protein to the *pac* region. However, the affinity is so weak (>100 μM) that as previously suggested (17,69) the sequence would be outcompeted by the vast number of non-specific sites on longer DNA. Thus, non-specific interactions would likely still dominate in the binding of small terminase to *pac* DNA.

### Characterization of the non-sequence-specific DNA binding activity of SF6 DBD

Since non-specific DNA binding appeared to be a major mode of interaction between DBD and DNA, the interaction was characterized further using complementary biophysical methods.

#### Surface plasmon resonance

**Figure 5.**
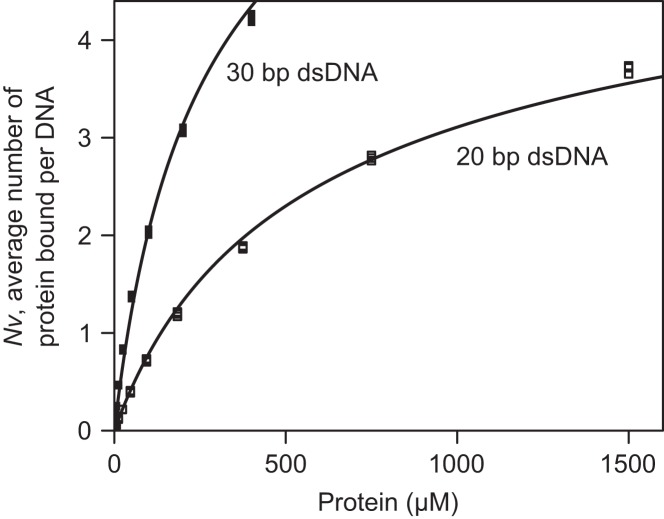
SPR analyses of SF6 DBD non-specific DNA-binding activities under the Tsodikov-corrected McGhee–von Hippel model. Protein (0.049–1500 μM) was injected over surface-immobilized 20-bp and 30-bp dsDNA ligands, respectively. SPR responses at equilibrium (dots) were double referenced against an unmodified surface and buffer injection controls, and the model fitted (lines).

Given similar refractive index increments, the relative masses of DNA immobilized and protein bound can be inferred from the SPR responses following DNA immobilization (*R*_L_) and at equilibrium when protein is injected (*R*_eq_). This enables analysis of non-sequence-specific binding activity under the (Tsodikov-modified) McGhee–von Hippel model (39,40). Under this model, DBD bound 20 and 30 bp DNA molecules with a site size of 3 bp and equilibrium dissociation constants, *K*_d_, of 0.92 ± 0.01 mM and 1.85 ± 0.02 mM, respectively (Figure 5 and Supplementary Figure S3). Note that these values are estimates as the system could not be saturated under the experimental conditions. In addition, artefacts might arise due to partial occlusion of the DNA as a result of surface immobilization, and partial binding of protein to DNA ends. In light of these uncertainties the binding of SF6 DBD to DNA was also analysed by AUC.

#### Analytical ultracentrifugation

**Figure 6.**
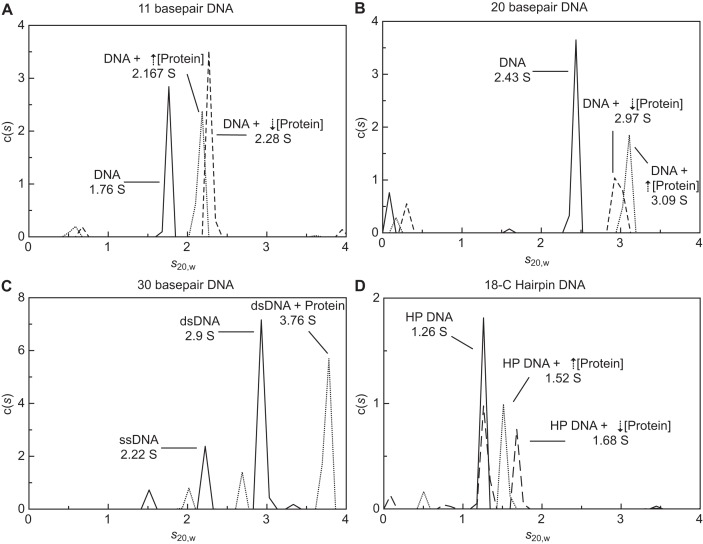
Sedimentation velocity profiles of SF6 DBD binding to (**A**) 11 bp, (**B**) 20 bp, (**C**) 30 bp DNA and (**D**) a model single-site DNA substrate (18C-hairpin, 5-bp DNA connected between strands by an 18C spacer on one end). Sedimentation profiles for each species are displayed as solid lines for DNA alone, and dashed or dotted lines for low and high concentrations of protein mixed with DNA, respectively. Further data and fitting information is provided in the Supplementary Material.

Sedimentation velocity AUC is a powerful tool for the analysis of multiple interacting and non-interacting species in a mixture of macromolecules (50,51). More recently, the technique has been extended to study rapidly reversible interactions and the assembly of protein–nucleic acid complexes (45–47). Here, sedimentation velocity AUC was used to characterize SF6 DBD in complex with different lengths of DNA (11, 20 and 30 bp; Supplementary Table S1; Supplementary Figure S4). The stoichiometries of fully bound complexes and average site size for DBD on DNA were determined (Supplementary Tables S6, S7; Figure 6). Note that the 30 bp DNA was derived from sequences covered by SPP1 *pac* site segments P4–P8.

So that DNA could be monitored independently of protein by absorption spectroscopy, DNA substrates were labelled on one strand with a fluorophore. Sedimentation profiles for dsDNA alone were used to define the sedimentation coefficient (*s_20,w_*) and frictional ratio (*f*/*f*_0_) for each DNA length (Figure 6; Supplementary Table S6). As the length of DNA increased, the respective values for *s_20,w_* and *f*/*f*_0_ also increased (Figure 6A–C; Supplementary Tables S5 and S6), reflecting the increase in molecular weight and the more extended shape of the DNA. Assuming a partial specific volume ($\overline \nu$) of 0.56 (for DNA in 100 mM KCl; (52)), molecular weights of 7.4, 13.5 and 21.4 kDa were determined for 11, 20 and 30 bp dsDNA, respectively, consistent with the predicted molecular weights of 7.5, 13.4 and 20.9 kDa, respectively (Supplementary Table S7). A peak corresponding to the labelled 30-nt ssDNA strand (∼20% of the total loaded signal) was observed and determined to have a molecular weight of 14.1 kDa, comparable to the predicted value of 10.7 kDa. Note that this variation in molecular weight is likely due to the fact that the *f*/*f*_0_ was fitted as an average over both ss and ds DNA species, hence the value for ssDNA is slightly higher and is reflected in the increased estimation for the molecular weight of this species (see SM).

Analysis of the sedimentation profiles for each DNA length with two concentrations of protein revealed a shift in the *s_20,w_* compared to the DNA alone, suggesting that a protein–DNA complex had formed in all cases (Figure 6A–C and Supplementary Table S7). The *f*/*f*_0_ value also increased compared to that for the DNA alone, indicating formation of a less flexible and more rod-like species (Supplementary Table S6). Since the conversion from *s_20,w_* to molecular weight for each protein–DNA complex containing different numbers of protein molecules was sensitive to the $\overline \nu$ parameter, that itself was dependent on the composition of the complex, conversion calculations were made for each potential protein bound state and then compared with the predicted molecular weights for each state (Supplementary Table S7). Since no unbound DNA was observed for both samples of the 11 bp DNA that contained protein, and the peaks corresponding to protein–DNA complex occur at similar *S*-values (Figure 6A), the 11 bp DNA was likely to be saturated with protein which produced a molecular weight of 23 and 31 kDa for each sample (Supplementary Table S7). This represented 2 and 3 proteins bound per 11 bp DNA and a site size of 4–5 bp. The 20 bp DNA formed complexes at low and high protein concentrations that likely represented different averages of rapidly re-associating partially bound (broad lower peak) and saturated complexes (narrower peak at higher *S*-value; Figure 6B). These represented complexes of approximately 39 and 48 kDa, consistent with predicted molecular weights for species with 3–4 and 5 proteins bound for an average site size of 4–5 bp (Supplementary Table S7). The 30 bp DNA species did not reach saturation upon addition of protein, as a peak corresponding to unbound DNA was also observed (Figure 6C). A larger species was observed at 3.76 S (Figure 6C), corresponding to a molecular weight of 60.4 kDa and consistent with a complex of DNA with 5 DBD molecules bound (Supplementary Table S7). Given that this species is not fully saturated, the site size can be determined as having an upper limit of 6 bp. These data are consistent with the SPR data above and additionally suggest that SF6 DBD binds *pac* and non-*pac* sequences in a similar, non-specific manner.

### Mutational analyses of SF6 DBD in *pac* site binding

Superposition of the structure of DBD with other HTH-containing proteins indicated several positively charged residues that might play a role in DNA binding (Figure 2). Of note, Lys48 in SF6 DBD is positioned similarly to Arg423 in hTRF1, which directly contacts a specific base of human telomeric DNA, and to Lys180 in PrrA, which occurs on a surface implicated in both specific and non-specific DNA interactions. Accordingly, Lys48 was mutated to an alanine residue, with no significant change in secondary structure content, and this led to an abrogation of DNA binding in both electromobility shift assays and SPR equilibrium assays (Supplementary Figure S5, 7), confirming that the residue forms part of the DNA-binding surface of SF6 DBD.

**Figure 7.**
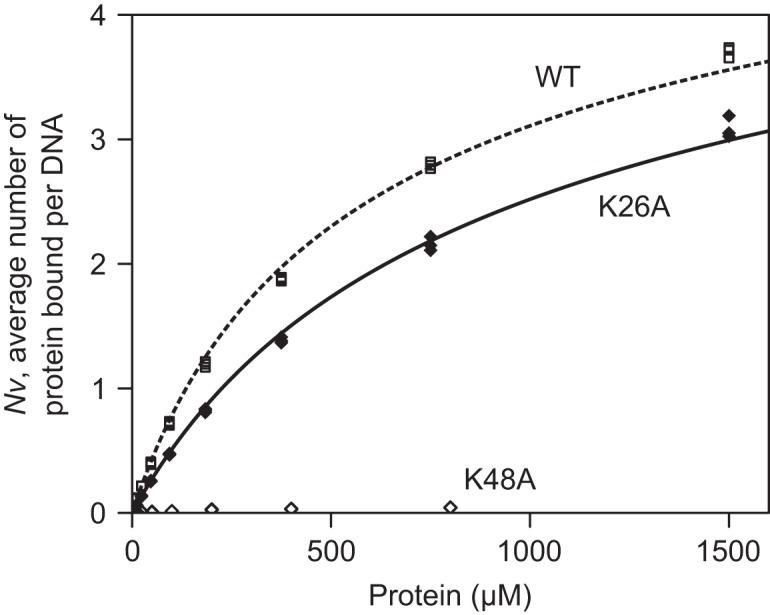
SPR equilibrium analyses of SF6 DBD K48A and K26A mutants. Protein (0.049–1500 μM) was injected over surface-immobilized 20-bp dsDNA ligand. Fits to K26A mutant and wild-type protein binding data are represented as solid and dotted lines, respectively.

Thr25/Lys26 of SF6 DBD occupy a similar position to Ser404/Lys405 in hTRF1, in the vicinity of the observed protein-DNA interface. Because a corresponding double mutation in the SPP1 small terminase reduces packaging efficiency *in vivo* (5), we speculated that a positive charge provided by Lys26 could contribute to the non-specific DNA-binding behaviour observed. However, on mutation to alanine, the protein bound DNA only a little less strongly with a *K*_d_ of 3.08 ± 0.03 mM and site size of 3 bp (under the Tsodikov-modified McGhee–von Hippel model), suggesting that Lys26 was in fact not directly involved in DNA binding and might contribute only to the overall electrostatics of the molecule.

### Design of a model single-site dsDNA substrate

Structural efforts to analyse the DBD-DNA interface at ambient temperatures have proven difficult in the past. As the SPR and AUC data reveal, the length of DNA required to form a stably annealed dsDNA substrate was such that the DNA would be long enough for more than one DBD molecule to bind, introducing heterogeneity. This heterogeneity was highlighted in chemical shift perturbation observed when 20-bp dsDNA was titrated into ^15^N-labelled protein. Signals were lost for all but the disordered regions of the protein (Supplementary Figure S6A), most likely due to complex size and exchange on the intermediate NMR timescale between complexes of different stoichiometries.

With an aim to reducing the system to a 1:1 protein–dsDNA interaction, model single-site substrates were designed. The substrates contained a 5-bp ds region, connected 3′-to-5′ by a 18C spacer (18C-hairpin) or 4-nt spacer (14-nt hairpin, Supplementary Table S1). The linkers would help the DNA remain annealed at room temperature. Competition SPR assays, where protein mixed with increasing amounts of 18C-hairpin were injected over surface-bound 30-bp DNA, confirmed that these hairpins could serve as substrates for DBD (Supplementary Figure S7). Sedimentation velocity AUC experiments were also conducted to assess complex stoichiometry. Analysis of the sedimentation profiles for 18C-hairpin alone yielded values of 1.3 S and 1.33 for *s_20,w_* and *f*/*f*_0_, respectively (Figure 6D; Supplementary Table S6). The calculated molecular weight of 4.3 kDa was comparable to the predicted 3.91 kDa (Supplementary Table S7). Mixing 5 μM of this hairpin and 1.5 mM protein gave rise to a single larger species with *s_20,w_* value of 1.56 S (Figure 6D), which corresponded to a molecular weight of 10.1 kDa, similar to the predicted value of 12.16 kDa for a 1:1 complex (Supplementary Table S7). At a lower protein concentration about half of the DNA was unbound while the rest was part of a larger species, also consistent with a 1:1 protein–DNA complex (Figure 6D, Supplementary Table S7, SM). Similar data were obtained using the 14-nt hairpin (Supplementary Figure S7). These affirmed that DBD would bind 5 bp of DNA 1:1 with a *K*_d_ in the mM range.

### The N-terminus and HTH motif of SF6 DBD mediate binding to a model DNA substrate

To characterize the DNA-binding surface of DBD in more detail, ^1^H, ^13^C and ^15^N NMR assignments were obtained for the backbone atoms of all residues except the first three and proline residues. Sidechain amide resonances were assigned for Asn36, Asn45 and Asn63.

**Figure 8.**
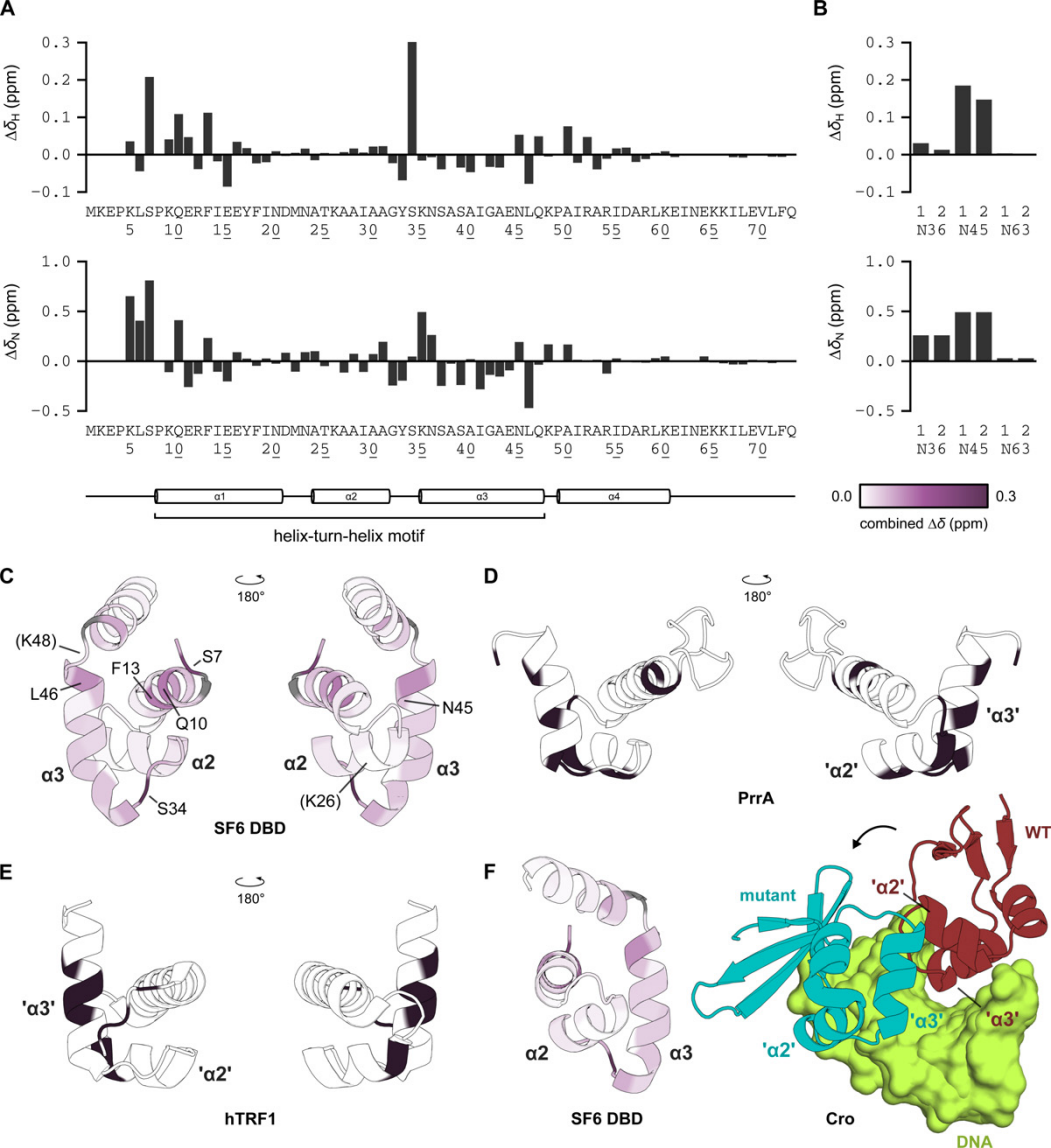
NMR analysis of the SF6 DBD DNA-binding surface. ^1^H and ^15^N chemical shift differences in (**A**) backbone and (**B**) sidechain amide groups in ^1^H-^15^N HSQC spectra on titration of 14-nt hairpin DNA into DBD, to a final molar ratio of 19.2:1. (**C**) Cartoon representations of SF6 DBD. Colours correspond to combined chemical shift difference magnitudes (see Supplementary Figure S8). Residues with differences more than one standard deviation in magnitude from the mean (>0.096 ppm), and residues K48, shown by mutagenesis to be important for DNA binding, and K26, which did not appear essential, (in brackets), are indicated. Unassigned residues are coloured grey. Cartoon representations of (**D**) PrrA and (**E**) hTRF1. Dark colour indicates residues showing significant chemical shift perturbation on interaction with DNA (61), and observed at the crystallographic protein–DNA interface (60), respectively. (**F**) The protein–DNA interfaces of specific-binding (WT, red) and non-specific-binding (mutant, blue) lambda Cro (PDB ID: 3ORC, 6CRO, respectively). The contributions of helices α2, α3 and the α2–α3 turn are altered on repositioning of the protein, and are broadly similar to the observed chemical shift difference pattern for SF6 DBD.

The 5-bp-forming 14-nt hairpin single-site DNA substrate was titrated into ^15^N-labelled sample and chemical shift differences measured (Figure 8A, B and Supplementary Figure S6B). The largest chemical shift differences occurred at the N-terminus and HTH motif (α1–3). These lie on the same face, coincide with the positively charged surface of the protein and map well to the solvent-accessible surface of the full-length oligomer (Figure 8C and Supplementary Figure S9). This implies that DBD interacts with DNA using a defined surface.

Chemical shift differences in the N-terminus suggest an N-terminal arm which binds DNA, akin to most HTH-containing proteins (70). Within the HTH motif, the α2–α3 turn and N-terminus of helix α3 were most affected by interaction with DNA, whereas residues in helix α2 exhibited little change in chemical shift. These data are at odds with what has been observed for proteins of the same HTH subfamily, where helix α3 would insert into the major groove and α2 would engage in steric, hydrogen bond and ion-dipole interactions with the DNA backbone (Figure 8D and E). Compared to PrrA, for which backbone chemical shift perturbation data on non-specific interaction are available (61), there are fewer strong shift differences in helix α2, relative to the rest of the protein and more in the α2–α3 turn. Looking broadly, however, this apparently different set of interacting or affected residues is not unique among HTH-containing proteins. When lambda Cro was engineered to bind DNA non-specifically, the interface became rotated (Figure 8F); the helix corresponding to α3 of SF6 DBD was more solvent-exposed and only the α2–α3 turn and N-terminus of α3 remained in contact with DNA (71,72). More detailed analyses would be necessary to discriminate between direct contacts and binding-induced conformational changes, but our current perturbation data suggest that the SF6 HTH motif does share similarities to a subset of HTH-containing proteins when interacting non-specifically with DNA.

## DISCUSSION

Specific recognition of *pac* DNA by small terminase is essential for initiating genome packaging during the assembly process of many bacteriophages. However, the mechanism by which this occurs is poorly defined. Previous work determined that the HTH motif in the N-terminal domain (DBD) of *Bacillus* phage SF6 small terminase was necessary for *pac* site binding (14), and it was speculated, due to the presence of repeat elements within the *pac* site, that the SF6 HTH motif is recruited to *pac* via a specific sequence. Our analyses by SPR, AUC and NMR showed that while SF6 DBD did interact with DNA, the observed interactions (Figures 3, 5, 6 and Supplementary Figure S6A) had the hallmarks of non-sequence-specific DNA binding: low affinity, rapid kinetics, and superstoichiometric binding (17). Further characterizing this binding mode, chemical shift perturbation data using a model substrate suggested that when presented with an arbitrary sequence the protein binds DNA in a way broadly similar to how HTH-containing transcription factors bind non-cognate sequences.

A small but weak preference for 5′-TTAGGTT-3′ was observed at high KCl concentrations (Figure 4). The identified sequence did not correlate with any of the box repeats in the SPP1 genome (20,22,23), suggesting that the repeats had little role in the nucleation of small terminase on *pac* DNA. However, even assuming a 1:1 interaction, the apparent affinity to the identified sequence (∼350 μM) is not substantially higher than the non-specific background (∼2 mM under the McGhee–von Hippel model). Given that the binding kinetics were similar, and considering that non-specific sites are available in much greater concentrations, it is unlikely that binding at this sequence is the main driver of complex assembly.

HTH motifs usually feature in DNA-binding assemblies as dimers or multiples of dimers. A dimeric structure enables the targeting of cognate sequence repeats separated by a defined distance, enhancing affinity and specificity. Of the known HTH motifs, some are capable of high-affinity interactions with DNA as a monomer, owing to additional structural elements (73–75); others, which the small terminase DBD resembles, bind DNA poorly as a monomer. However, given the multimeric nature of the latter motifs, measuring their affinity as a monomer for DNA has not been trivial. Model systems have been created, with mutations or truncations in the DNA substrate, to simulate monomeric interactions. These systems have produced estimates of 10–100 nM (hTRF1 (19); c-myb (64)), 2–4 μM (Cro (65)) affinities for a single-site interaction. Our data suggest that individual HTH motifs of the small terminase bind with even lower affinity.

Thus, we propose that the small terminase proteins from *pac* phages use a different strategy for selecting DNA. HTH motifs are employed in great numbers. Each motif has low, milimolar affinity, with slight preference for a sequence which occurs only one to three times in the cognate region. Small terminase may be recruited to *pac* DNA through this sequence, but stabilization of the protein–DNA complex is more likely dependent on sequence-independent associations through multiple HTH motifs. A DNA segment such as the *pac* region, which easily conforms to the circular arrangement of HTH motifs, would favour such associations. DNA is, therefore, selected by conformation. Such ‘conformational selection’ is reminiscent of interactions that drive the assembly of eukaryotic nucleosome core particles (76,77). Indeed, the DNA sequence content and mapping of *pac* DNA is similar to well-characterized nucleosome core particle sequences (76–79), where runs of 5–7 AT base pairs are interspersed with double GC or CG pairs. These sequences enable bending and kinking, favouring ∼100 bp helicoidal DNA segments which would be ideal for wrapping around small terminase.

A ‘conformational selection’ model is consistent with available binding data for SPP1-like viruses: that *pac* DNA had intrinsic flexibility and populated the bent state more frequently in the presence of small terminase (20,22,23). Here we show that one SF6 DBD occludes 3–5 bp of DNA. Multiple DBDs arranged circularly with defined spacing would reproduce the regularly spaced DNAse I protection patterns previously observed for SPP1 small terminase-*pac* (23) and eukaryotic nucleosome core particles (79).

Small terminase proteins from other viruses occur in similar oligomeric states as the SF6 small terminase, with HTH motifs displayed around a central core (Figure 1A) (11–14,80). The diameter and circumference of these oligomers are ∼90 and ∼300 Å, respectively, and adjacent HTH motifs are consistently separated by a distance approximate to one full turn of B-form DNA. The solvent-accessible surfaces of individual HTH motifs combine to form a characteristic positively charged belt (Supplementary Figure S10), permissive to a model where DNA is wrapped around small terminase. However, note that this mode of interaction may not be conserved across *pac* viruses. For example, it is not clear whether T4 small terminase proteins contain an N-terminal HTH motif (81), while the P22 HTH-like motif is occluded from the solvent by an N-terminal segment (13). Instead, both termini in T4 (82) and the C-terminus in P22 (80,83) are implicated in DNA binding.

**Figure 9.**
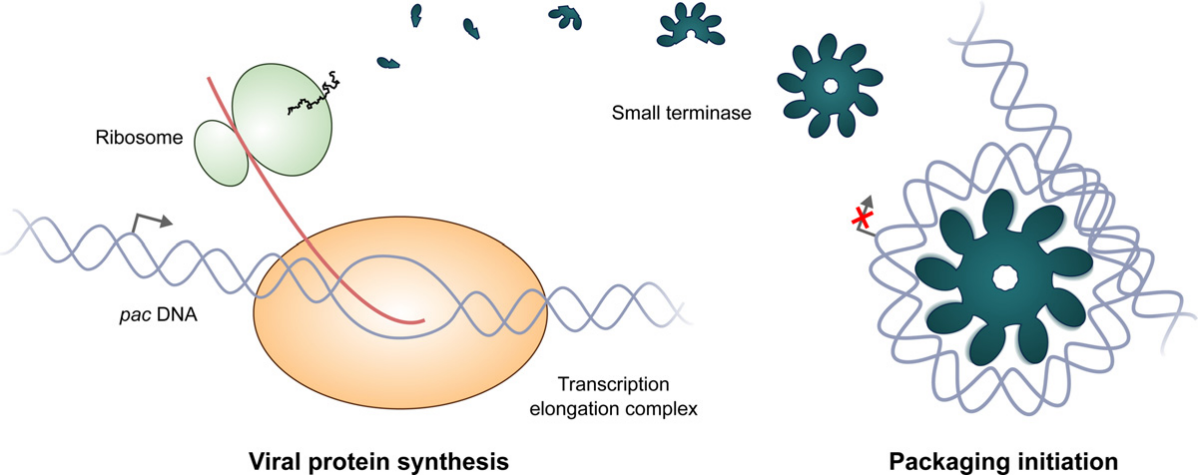
Small terminase as a switch for viral DNA packaging. Multiple, weak, non-specific HTH–DNA interactions in a circular array enable recognition of viral DNA for packaging, while mediating gene regulation and ensuring favourable stoichiometries between DNA copies and motor components.

The idea of conformational selection by an array of circularly arranged, weak, non-specific binders has several biological implications. In all domains of life the architecture of nucleoprotein complexes is exploited for the regulation of gene expression. In eukaryotes, nucleosome core particles (84) and in prokaryotes, H-NS, HU and similar proteins (85,86), prevent transcription initiation or elongation by the RNA polymerase. Similarly, the small terminase-*pac* DNA complex has been shown to downregulate transcription initiation of the motor protein operon, presumably by competition with RNA polymerase assembly in the promoter region (87). Under the proposed model, the large DNA footprint enables the protein to act as a switch between transcription and packaging initiation (Figure 9). Such regulation may help ensure favourable stoichiometries between genomic DNA copies and motor assemblies so that use of energy and resources within the host is efficient. In the context of DNA packaging, stabilization of a bent DNA conformation favours cleavage by large terminase (88). The use of multiple individually weak binders also confers reversibility. The transient and weak nature of the interactions between individual DBDs and DNA (Figures 3A and 5) means that DNA in complex with small terminase could be unwrapped under external forces such as during translocation.

Another major advantage of a conformation-selective complex is the high tolerance for DNA sequence variation in the recognition region. This allows for a more compact genome, as the region can take on multiple roles, in the case of SF6, accommodating the promoters, ribosomal binding sites and beginning of the small terminase gene-coding sequence. Such a phenomenon would reflect pressures to minimize genome size for efficient replication and packaging while maximizing coding density and function.

In conclusion, we propose that the small terminase protein of SPP1-like viruses represents a new class of HTH-containing DNA-binding proteins where the common HTH motif, which binds strongly to short specific DNA sequences, is adapted into an oligomeric circular array where individual HTH motifs form weak, predominantly non-specific interactions with DNA. The result is a robust packaging initiation system that also tolerates mutagenic drift, maintains minimal genome size and tightly regulates the switch between viral DNA replication and packaging.

## ACCESSION NUMBERS

The structure of the SF6 GP1 DBD is deposited under PDB ID 4ZC3.

## Supplementary Material

SUPPLEMENTARY DATA

## References

[1] Steitz T.A. (1990). Structural studies of protein-nucleic acid interaction: the sources of sequence-specific binding. Q. Rev. Biophys..

[2] Rohs R., Jin X., West S.M., Joshi R., Honig B., Mann R.S. (2010). Origins of specificity in protein-DNA recognition. Annu. Rev. Biochem..

[3] Casjens S.R. (2011). The DNA-packaging nanomotor of tailed bacteriophages. Nature Rev. Microbiol..

[4] Feiss M., Rao V.B. (2012). The bacteriophage DNA packaging machine. Adv. Exp. Med. Biol..

[5] Oliveira L., Tavares P., Alonso J.C. (2013). Headful DNA packaging: bacteriophage SPP1 as a model system. Virus Res..

[6] Lehman I.R., Boehmer P.E. (1999). Replication of herpes simplex virus DNA. J. Biol. Chem..

[7] de Beer T., Fang J., Ortega M., Yang Q., Maes L., Duffy C., Berton N., Sippy J., Overduin M., Feiss M. (2002). Insights into specific DNA recognition during the assembly of a viral genome packaging machine. Mol. Cell.

[8] Jackson E.N., Jackson D.A., Deans R.J. (1978). *Eco*RI analysis of bacteriophage P22 DNA packaging. J. Mol. Biol..

[9] Adams M.B., Hayden M., Casjens S. (1983). On the sequential packaging of bacteriophage P22 DNA. J. Virol..

[10] Tavares P., Lurz R., Stiege A., Ruckert B., Trautner T.A. (1996). Sequential headful packaging and fate of the cleaved DNA ends in bacteriophage SPP1. J. Mol. Biol..

[11] Zhao H., Finch C.J., Sequeira R.D., Johnson B.A., Johnson J.E., Casjens S.R., Tang L. (2010). Crystal structure of the DNA-recognition component of the bacterial virus Sf6 genome-packaging machine. Proc. Natl. Acad. Sci. U.S.A..

[12] Zhao H., Kamau Y.N., Christensen T.E., Tang L. (2012). Structural and functional studies of the phage Sf6 terminase small subunit reveal a DNA-spooling device facilitated by structural plasticity. J. Mol. Biol..

[13] Roy A., Bhardwaj A., Datta P., Lander G.C., Cingolani G. (2012). Small terminase couples viral DNA binding to genome-packaging ATPase activity. Structure.

[14] Büttner C.R., Chechik M., Ortiz-Lombardia M., Smits C., Ebong I.O., Chechik V., Jeschke G., Dykeman E., Benini S., Robinson C.V. (2012). Structural basis for DNA recognition and loading into a viral packaging motor. Proc. Natl. Acad. Sci. U.S.A..

[15] Aravind L., Anantharaman V., Balaji S., Babu M.M., Iyer L.M. (2005). The many faces of the helix-turn-helix domain: transcription regulation and beyond. FEMS Microbiol. Rev..

[16] Muller C.W. (2001). Transcription factors: global and detailed views. Curr. Opin. Struct. Biol..

[17] Oda M., Nakamura H. (2000). Thermodynamic and kinetic analyses for understanding sequence-specific DNA recognition. Genes Cells.

[18] Laguri C., Stenzel R.A., Donohue T.J., Phillips-Jones M.K., Williamson M.P. (2006). Activation of the global gene regulator PrrA (RegA) from Rhodobacter sphaeroides. Biochemistry.

[19] Konig P., Fairall L., Rhodes D. (1998). Sequence-specific DNA recognition by the myb-like domain of the human telomere binding protein TRF1: a model for the protein-DNA complex. Nucleic Acids Res..

[20] Chai S., Bravo A., Luder G., Nedlin A., Trautner T.A., Alonso J.C. (1992). Molecular analysis of the *Bacillus subtilis* bacteriophage SPP1 region encompassing genes 1 to 6. The products of gene 1 and gene 2 are required for pac cleavage. J. Mol. Biol..

[21] Chai S., Kruft V., Alonso J.C. (1994). Analysis of the *Bacillus subtilis* bacteriophages SPP1 and SF6 gene 1 product: a protein involved in the initiation of headful packaging. Virology.

[22] Chai S., Lurz R., Alonso J.C. (1995). The small subunit of the terminase enzyme of *Bacillus subtilis* bacteriophage SPP1 forms a specialized nucleoprotein complex with the packaging initiation region. J. Mol. Biol..

[23] Gual A., Alonso J.C. (1998). Characterization of the small subunit of the terminase enzyme of the *Bacillus subtilis* bacteriophage SPP1. Virology.

[24] Gill S.C., von Hippel P.H. (1989). Calculation of protein extinction coefficients from amino acid sequence data. Anal. Biochem..

[25] Pace C.N., Vajdos F., Fee L., Grimsley G., Gray T. (1995). How to measure and predict the molar absorption coefficient of a protein. Protein Sci..

[26] Gray D.M., Hung S.H., Johnson K.H. (1995). Absorption and circular dichroism spectroscopy of nucleic acid duplexes and triplexes. Methods Enzymol..

[27] Studier F.W. (2005). Protein production by auto-induction in high density shaking cultures. Protein Expr. Purif..

[28] Muona M., Aranko A.S., Iwai H. (2008). Segmental isotopic labelling of a multidomain protein by protein ligation by protein trans-splicing. Chembiochem.

[29] McCoy A.J., Grosse-Kunstleve R.W., Adams P.D., Winn M.D., Storoni L.C., Read R.J. (2007). Phaser crystallographic software. J. Appl. Crystallogr..

[30] Yao J.X., Woolfson M.M., Wilson K.S., Dodson E.J. (2005). A modified ACORN to solve protein structures at resolutions of 1.7 Å or better. Acta Crystallogr. D Biol. Crystallogr..

[31] Langer G., Cohen S.X., Lamzin V.S., Perrakis A. (2008). Automated macromolecular model building for X-ray crystallography using ARP/wARP version 7. Nat. Protoc..

[32] Emsley P., Lohkamp B., Scott W.G., Cowtan K. (2010). Features and development of Coot. Acta Crystallogr. D Biol. Crystallogr..

[33] Murshudov G.N., Vagin A.A., Dodson E.J. (1997). Refinement of macromolecular structures by the maximum-likelihood method. Acta Crystallogr. D Biol. Crystallogr..

[34] Painter J., Merritt E. (2005). TLSMD web server for the generation of multi-group TLS models. J. Appl. Crystallogr..

[35] Krissinel E., Henrick K. (2004). Secondary-structure matching (SSM), a new tool for fast protein structure alignment in three dimensions. Acta Crystallogr. D Biol. Crystallogr..

[36] McNicholas S., Potterton E., Wilson K.S., Noble M.E. (2011). Presenting your structures: the CCP4mg molecular-graphics software. Acta Crystallogr. D Biol. Crystallogr..

[37] Myszka D.G. (1999). Improving biosensor analysis. J. Mol. Recognit..

[38] Stevenson C.E., Assaad A., Chandra G., Le T.B., Greive S.J., Bibb M.J., Lawson D.M. (2013). Investigation of DNA sequence recognition by a streptomycete MarR family transcriptional regulator through surface plasmon resonance and X-ray crystallography. Nucleic Acids Res..

[39] McGhee J.D., von Hippel P.H. (1974). Theoretical aspects of DNA-protein interactions: Co-operative and non-co-operative binding of large ligands to a one-dimensional homogeneous lattice. J. Mol. Biol..

[40] Tsodikov O.V., Holbrook J.A., Shkel I.A., Record M.T. (2001). Analytic binding isotherms describing competitive interactions of a protein ligand with specific and nonspecific sites on the same DNA oligomer. Biophys. J..

[41] di Primo C., Lebars I. (2007). Determination of refractive index increment ratios for protein–nucleic acid complexes by surface plasmon resonance. Anal. Biochem..

[42] R Core Team (2013). R: A language and environment for statistical computing.

[43] Byrd R., Lu P., Nocedal J., Zhu C. (1995). A limited memory algorithm for bound constrained optimization. SIAM J. Sci. Stat. Comput..

[44] Cole J.L., Lary J.W., Moody T., Laue T.M. (2008). Analytical ultracentrifugation: sedimentation velocity and sedimentation equilibrium. Methods Cell Biol..

[45] Greive S.J., Lins A.F., von Hippel P.H. (2005). Assembly of an RNA-protein complex. Binding of NusB and NusE (S10) proteins to boxA RNA nucleates the formation of the antitermination complex involved in controlling rRNA transcription in *Escherichia coli*. J. Biol. Chem..

[46] Eustermann S., Videler H., Yang J.C., Cole P.T., Gruszka D., Veprintsev D., Neuhaus D. (2011). The DNA-binding domain of human PARP-1 interacts with DNA single-strand breaks as a monomer through its second zinc finger. J. Mol. Biol..

[47] Jose D., Weitzel S.E., Jing D., von Hippel P.H. (2012). Assembly and subunit stoichiometry of the functional helicase-primase (primosome) complex of bacteriophage T4. Proc. Natl. Acad. Sci. U.S.A..

[48] Laue T., Shah B., Ridgeway T., Pelletier S., Harding S, Rowe A, Horton J (1992). Computer-aided interpretation of analytical sedimentation data for proteins. Analytical Ultracentrifugation in Biochemistry and Polymer Science.

[49] Schuck P., MacPhee C.E., Howlett G.J. (1998). Determination of sedimentation coefficients for small peptides. Biophys. J..

[50] Schuck P., Scott D, Harding S, Rowe A (2005). Diffusion-deconvoluted sedimentation coefficient distributions for the analysis of interacting and non-interacting protein mixtures. Analytical Ultracentrifugation: Techniques and Methods.

[51] Schuck P. (2010). Sedimentation patterns of rapidly reversible protein interactions. Biophys. J..

[52] Bonifacio G.F., Brown T., Conn G.L., Lane A.N. (1997). Comparison of the electrophoretic and hydrodynamic properties of DNA and RNA oligonucleotide duplexes. Biophys. J..

[53] Perkins S.J. (1986). Protein volumes and hydration effects. The calculations of partial specific volumes, neutron scattering matchpoints and 280-nm absorption coefficients for proteins and glycoproteins from amino acid sequences. Eur. J. Biochem..

[54] Durchschlag H., Hinz H (1986). Specific volumes of biological macromolecules and some other molecules of biological interest. Thermodynamic Data for Biochemistry and Biotechnology.

[55] Orekhov V.Y., Jaravine V.A. (2011). Analysis of non-uniformly sampled spectra with multi-dimensional decomposition. Prog. Nucl. Magn. Reson. Spectrosc..

[56] Delaglio F., Grzesiek S., Vuister G.W., Zhu G., Pfeifer J., Bax A. (1995). NMRPipe: a multidimensional spectral processing system based on UNIX pipes. J. Biomol. NMR.

[57] Vranken W.F., Boucher W., Stevens T.J., Fogh R.H., Pajon A., Llinas M., Ulrich E.L., Markley J.L., Ionides J., Laue E.D. (2005). The CCPN data model for NMR spectroscopy: development of a software pipeline. Proteins.

[58] Williamson M.P. (2013). Using chemical shift perturbation to characterise ligand binding. Prog. Nucl. Magn. Reson. Spectrosc..

[59] Benini S., Chechik M., Ortiz Lombardia M., Polier S., Leech A., Shevtsov M.B., Alonso J.C. (2013). The 1.58 Å resolution structure of the DNA-binding domain of bacteriophage SF6 small terminase provides new hints on DNA binding. Acta Crystallogr. F Struct. Biol. Commun..

[60] Court R., Chapman L., Fairall L., Rhodes D. (2005). How the human telomeric proteins TRF1 and TRF2 recognize telomeric DNA: a view from high-resolution crystal structures. EMBO Rep..

[61] Laguri C., Phillips-Jones M.K., Williamson M.P. (2003). Solution structure and DNA binding of the effector domain from the global regulator PrrA (RegA) from *Rhodobacter sphaeroides*: insights into DNA binding specificity. Nucleic Acids Res..

[62] Hanaoka S., Nagadoi A., Nishimura Y. (2005). Comparison between TRF2 and TRF1 of their telomeric DNA-bound structures and DNA-binding activities. Protein Sci..

[63] Eraso J.M., Kaplan S. (2009). Half-site DNA sequence and spacing length contributions to PrrA binding to PrrA site 2 of RSP3361 in *Rhodobacter sphaeroides* 2.4.1. J. Bacteriol..

[64] Oda M., Furukawa K., Sarai A., Nakamura H. (1999). Kinetic analysis of DNA binding by the c-Myb DNA-binding domain using surface plasmon resonance. FEBS Lett..

[65] Baleja J.D., Anderson W.F., Sykes B.D. (1991). Different interactions of Cro repressor dimer with the left and right halves of OR3 operator DNA. J. Biol. Chem..

[66] Lohman T.M., DeHaseth P.L., Record M.T. (1978). Analysis of ion concentration effects of the kinetics of protein-nucleic acid interactions. Application to *lac* repressor-operator interactions. Biophys. Chem..

[67] Bailey T.L., Boden M., Buske F.A., Frith M., Grant C.E., Clementi L., Ren J., Li W.W., Noble W.S. (2009). MEME SUITE: tools for motif discovery and searching. Nucleic Acids Res..

[68] Grant C.E., Bailey T.L., Noble W.S. (2011). FIMO: scanning for occurrences of a given motif. Bioinformatics.

[69] von Hippel P.H., Berg O.G. (1986). On the specificity of DNA-protein interactions. Proc. Natl. Acad. Sci. U.S.A..

[70] Eliason J.L., Weiss M.A., Ptashne M. (1985). NH2-terminal arm of phage lambda repressor contributes energy and specificity to repressor binding and determines the effects of operator mutations. Proc. Natl. Acad. Sci. U.S.A..

[71] Albright R.A., Matthews B.W. (1998). Crystal structure of lambda-Cro bound to a consensus operator at 3.0 Å resolution. J. Mol. Biol..

[72] Albright R.A., Mossing M.C., Matthews B.W. (1998). Crystal structure of an engineered Cro monomer bound nonspecifically to DNA: possible implications for nonspecific binding by the wild-type protein. Protein Sci..

[73] Gehring W.J., Affolter M., Burglin T. (1994). Homeodomain proteins. Annu. Rev. Biochem..

[74] Gajiwala K.S., Burley S.K. (2000). Winged helix proteins. Curr. Opin. Struct. Biol..

[75] Himes P., McBryant S.J., Kroos L. (2010). Two regions of *Bacillus subtilis* transcription factor SpoIIID allow a monomer to bind DNA. J. Bacteriol..

[76] Ong M.S., Richmond T.J., Davey C.A. (2007). DNA stretching and extreme kinking in the nucleosome core. J. Mol. Biol..

[77] Trifonov E.N. (2011). Cracking the chromatin code: precise rule of nucleosome positioning. Phys. Life Rev..

[78] Rohs R., West S.M., Sosinsky A., Liu P., Mann R.S., Honig B. (2009). The role of DNA shape in protein-DNA recognition. Nature.

[79] Prunell A., Kornberg R.D., Lutter L., Klug A., Levitt M., Crick F.H. (1979). Periodicity of deoxyribonuclease I digestion of chromatin. Science.

[80] Nemecek D., Lander G.C., Johnson J.E., Casjens S.R., Thomas G.J. Jr. (2008). Assembly architecture and DNA binding of the bacteriophage P22 terminase small subunit. J. Mol. Biol..

[81] Sun S., Gao S., Kondabagil K., Xiang Y., Rossmann M.G., Rao V.B. (2012). Structure and function of the small terminase component of the DNA packaging machine in T4-like bacteriophages. Proc. Natl. Acad. Sci. U.S.A..

[82] Black L.W. (2015). Old, new, and widely true: The bacteriophage T4 DNA packaging mechanism. Virology.

[83] Wu H., Sampson L., Parr R., Casjens S. (2002). The DNA site utilized by bacteriophage P22 for initiation of DNA packaging. Mol. Microbiol..

[84] Venkatesh S., Workman J.L. (2015). Histone exchange, chromatin structure and the regulation of transcription. Nat. Rev. Mol. Cell Biol..

[85] Dorman C.J. (2004). H-NS: a universal regulator for a dynamic genome. Nat. Rev. Microbiol..

[86] Lewis D.E., Geanacopoulos M., Adhya S. (1999). Role of HU and DNA supercoiling in transcription repression: specialized nucleoprotein repression complex at gal promoters in *Escherichia coli*. Mol. Microbiol..

[87] Chai S., Szepan U., Alonso J.C. (1997). *Bacillus subtilis* bacteriophage SPP1 terminase has a dual activity: it is required for the packaging initiation and represses its own synthesis. Gene.

[88] Gual A., Camacho A.G., Alonso J.C. (2000). Functional analysis of the terminase large subunit, G2P, of *Bacillus subtilis* bacteriophage SPP1. J. Biol. Chem..

