# Supplementary material for: DNA recognition for virus assembly through multiple sequence-independent interactions with a helix-turn-helix motif
Source: Nucleic Acids Res. 2015 Dec 15;44(2):776–89. doi: 10.1093/nar/gkv1467 (PMC4737164; doi:10.1093/nar/gkv1467)
Supplement: SUPPLEMENTARY DATA [file supp_44_2_776__index.html]

DNA recognition for virus assembly through multiple sequence-independent interactions with a helix-turn-helix motif — SUPPLEMENTARY DATA 

# DNA recognition for virus assembly through multiple sequence-independent interactions with a helix-turn-helix motif

## SUPPLEMENTARY DATA

- SUPPLEMENTARY DATA
