# Supplementary material for: DNA recognition for virus assembly through multiple sequence-independent interactions with a helix-turn-helix motif
Source: Nucleic Acids Res. 2015 Dec 15;44(2):776–89. doi: 10.1093/nar/gkv1467 (PMC4737164; doi:10.1093/nar/gkv1467)
Supplement: SUPPLEMENTARY DATA [file supp_gkv1467_nar-03248-f-2015-File011.pdf]

Supplementary Material for

DNA recognition for virus assembly through multiple sequence-independent interactions with a helix-turn-helix motif

Sandra J Greive^, Herman KH Fung^, Maria Chechik, Huw T Jenkins, Stephen E Weitzel, Pedro M Aguiar, Andrew S Brentnall, Matthieu Gloussieau, Grigory V Gladyshev, Jennifer R Potts and Alfred A Antson

Table S1. Oligonucleotide sequences for SPR equilibrium, AUC sedimentation velocity and NMR chemical shift perturbation studies.

Oligo	Experiment(s)	Sequence	Mass (kDa)	ε_{260} ($M^{-1} \cdot cm^{-1}$)
**5′-biotinylated 20-nt linker**	SPR	[BIO]-GCAGGAGGACGTAGGGTAGG	6.39	210500
**20-nt linker complement**	SPR, AUC, NMR	CCTACCCTACGTCCTCCTGC	5.92	166100
**5′-biotinylated 30-nt linker**	SPR	[BIO]-GAGACCTGGAACGCGATCATTGGCAGCACG	9.67	292600
**30-nt linker complement**	SPR	CGTGCTGCCAATGATCG CGTTCCAGGTCTC	9.15	269800
**fluorescent 20-nt linker**	AUC	GCAGGAGGACGTAGGGTAGG-[AT647N]	7.10	229200
**fluorescent 30-nt (AUC) DNA**	AUC	[TAMRA]-ATAGAAAAAACCTCCTTAAAACTATATTTG	9.76	336300
**30-nt (AUC) DNA complement**	AUC	CAAATATAGTTTTAAGGAGGTTTTTTCTAT	9.24	298000
**fluorescent 11-nt DNA**	AUC	GCCTACCCTAC-[AT647N]	4.04	114500
**11-nt DNA complement**	AUC	GTAGGGTAGGC	3.44	113500
**fluorescent 14-nt hairpin**	AUC	GTAGGTTTTCCTAC-[AT647N]	5.08	148900
**fluorescent 18C-hairpin**	AUC	[6-FAM]-AGTAGG-[18C]-CCTAC	4.22	130460
**14-nt hairpin**	SPR	GTAGGTTTTCCTAC	4.25	130200
**18C-hairpin**	SPR	AGTAGG-[18C]-CCTAC	3.69	109500
**20-nt linker**	NMR	GCAGGAGGACGTAGGGTAGG	6.15	210500

Table S2. Oligonucleotide sequences for ReDCaT assay. SPP1 and SF6 *pac* sites were screened in 30-bp segments, designated P# and S#, respectively, with 5-bp overlap on either end. The top and bottom strands of each segment are indicated. The '20-nt linker complement' sequence (in lowercase) was appended to the 3′ end of the bottom strand to enable annealing with surface-bound 5′-biotinylated 20-nt linker.

Oligo	Sequence
**P1 top**	CCGGCTGATGTTTTGAAAGAAGCGAAAAAG
**P1 bottom**	CTTTTTCGCTTCTTTCAAAACATCAGCCGGcctaccctacgtcctcctgc
**P2 top**	AAAAGCGGCGGTTATATCTTGTAGCGTAGA
**P2 bottom**	TCTACGCTACAAGATATAACCGCCGCTTTTcctaccctacgtcctcctgc
**P3 top**	GTAGAATGTTATAATGGATTTAATTGGCGG
**P3 bottom**	CCGCCAATTAAATCCATTATAACATTCTACcctaccctacgtcctcctgc
**P4 top**	GGCGGAATAACACAGAGAGGCACCCTATTT
**P4 bottom**	AAATAGGGTGCCTCTCTGTGTTATTCCGCCcctaccctacgtcctcctgc
**P5 top**	TATTTGGGTGCTTTTTTGTTGTATAATTAG
**P5 bottom**	CTAATTATACAACAAAAAAGCACCCAAATAcctaccctacgtcctcctgc
**P6 top**	ATTAGGTTTATATAAGGTTTTATCATTCAG
**P6 bottom**	CTGAATGATAAAACCTTATATAAACCTAATcctaccctacgtcctcctgc
**P7 top**	TTCAGATATGAGGTTCAAATATAGTTTTAA
**P7 bottom**	TTAAAACTATATTTGAACCTCATATCTGAAcctaccctacgtcctcctgc
**P8 top**	TTTAAGGAGGTTTTTTCTATGGGAGAAGTA
**P8 bottom**	TACTTCTCCCATAGAAAAAACCTCCTTAAAcctaccctacgtcctcctgc
**P9 top**	AAGTAAAGGGGAAATGGACGCCCAAACTCG
**P9 bottom**	CGAGTTTGGGCGTCCATTTCCCCTTTACTTcctaccctacgtcctcctgc
**P10 top**	ACTCGAAAGATTTGTTGATGAATATTTCAT
**P10 bottom**	ATGAAATATTCATCAACAAATCTTTCGAGTcctaccctacgtcctcctgc
**P11 top**	TTCATAAACGGCATGAATGCAACAAAAGCG
**P11 bottom**	CGCTTTTGTTGCATTCATGCCGTTTATGAAcctaccctacgtcctcctgc

**P12 top**	AAGCGGCTATTGCGGCTGGTTATAGTAAAA
**P12 bottom**	TTTTACTATAACCAGCCGCAATAGCCGCTTcctaccctacgtcctcctgc
**P13 top**	TAAAAAGTCTGCTTCGACTATTGCGGCCGA
**P13 Comp**	TCGGCCGCAATAGTCGAAGCAGACTTTTTAcctaccctacgtcctcctgc
**P14 top**	GCCGAGAACATGCAAAAACCGCACGTCCGC
**P14 bottom**	GCGGACGTGCGGTTTTTGCATGTTCTCGGCcctaccctacgtcctcctgc
**P15 top**	TCCGCGCACGTATCGAGGAAAGATTGGCAC
**P15 bottom**	GTGCCAATCTTTCCTCGATACGTGCGCGGAcctaccctacgtcctcctgc
**P16 top**	AGATTGGCACAAATGGACAAGAAAAGAATC
**P16 bottom**	GATTCTTTTCTTGTCCATTTGTGCCAATCTcctaccctacgtcctcctgc
**S1 top**	GGCGGAATAACACAGAGAGGTACCCTATTT
**S1 bottom**	AAATAGGGTACCTCTCTGTGTTATTCCGCCcctaccctacgtcctcctgc
**S2 top**	TATTTGGGTGCTTTTTTTGTTGTATAATTAG
**S2 bottom**	CTAATTATACAACAAAAAAGCACCCAAATAcctaccctacgtcctcctgc
**S3 top**	ATTAGGTTTATATAAGGTTTTAATTAGGTT
**S3 bottom**	AACCTAATTAAAACCTTATATAAACCTAATcctaccctacgtcctcctgc
**S4 top**	AGGTTCAAATATAGTTAGGAGGGGATTTTA
**S4 bottom**	TAAAATCCCCTCCTAACTATATTTGAACCTcctaccctacgtcctcctgc
**S5 top**	TTTTATGAAAGAACCTAAACTATCTCCAAA
**S5 bottom**	TTTGGAGATAGTTTAGGTTCTTTCATAAAAcctaccctacgtcctcctgc
**S6 top**	CCAAAACAGGAAAGATTTATTGAAGAGTAT
**S6 bottom**	ATACTCTTCAATAAATCTTTCCTGTTTTGGcctaccctacgtcctcctgc
**S7 top**	AGTATTTTATAAACGACATGAACGCTACTA
**S7 bottom**	TAGTAGCGTTCATGTCGTTTATAAAATACTcctaccctacgtcctcctgc
**S8 top**	TACTAAAGCGGCTATTGCGGCAGGATATAG
**S8 bottom**	CTATATCCTGCCGCAATAGCCGCTTTAGTAcctaccctacgtcctcctgc
**S9 top**	TATAGTAAAAATTCCGCGTCTGCTATTGGG

**S9 bottom**	CCCAATAGCAGACGCGGAATTTTTACTATAcctaccctacgtcctcctgc
**S10 top**	TTGGGGCAGAAAACTTACAGAAACCAGCTA
**S10 bottom**	TAGCTGGTTTCTGTAAGTTTTCTGCCCCAAcctaccctacgtcctcctgc
**S11 top**	AGCTATCCGCGCACGTATTGACGCTCGTTT
**S11 bottom**	AAACGAGCGTCAATACGTGCGCGGATAGCTcctaccctacgtcctcctgc
**S12 top**	CGTTTGAAGGAAATAAACGAGAAGAAAATC
**S12 bottom**	GATTTTCTTCTCGTTTATTTCCTTCAAACGcctaccctacgtcctcctgc
**S13 top**	AAATCCTCCAAGCTAACGAGGTTTTGGAGC
**S13 bottom**	GCTCCAAAACCTCGTTAGCTTGGAGGATTTcctaccctacgtcctcctgc
**S14 top**	GGAGCATTTGACTCGCATTGCACTCGGCCA
**S14 bottom**	TGGCCGAGTGCAATGCGAGTCAAATGCTCCcctaccctacgtcctcctgc
**S15 top**	GGCCAGGAAAAGGAACAGGTGCTCATGGGA
**S15 bottom**	TCCCATGAGCACCTGTTCCTTTTCCTGGCCcctaccctacgtcctcctgc
**S16 top**	GGAAAAGGAACAGGTGCTCATGGGAATTGG
**S16 bottom**	CCAATTCCCATGAGCACCTGTTCCTTTTCCcctaccctacgtcctcctgc
**S14_L top**	TTGCGGCCGAGAACATGCAAAAACCGCACGTCCG
**P14_L bottom**	CGGACGTGCGGTTTTTGCATGTTCTCGGCCGCAAcctaccctacgtcctcctgc
**A20G top**	TTGCGGCCGAGAACATGCAGAAACCGCACGTCCG
**A20G bottom**	CGGACGTGCGGTTTCTGCATGTTCTCGGCCGCAAcctaccctacgtcctcctgc
**C4G top**	TTGGGGCCGAGAACATGCAGAAACCGCACGTCCG
**C4G bottom**	CGGACGTGCGGTTTCTGCATGTTCTCGGCCCCAAcctaccctacgtcctcctgc
**G11A top**	TTGGGGCCGAAAACATGCAGAAACCGCACGTCCG
**G11A bottom**	CGGACGTGCGGTTTCTGCATGTTTTCGGCCCCAAcctaccctacgtcctcctgc
**C8A top**	TTGGGGCAGAAAACATGCAGAAACCGCACGTCCG
**C8A bottom**	CGGACGTGCGGTTTCTGCATGTTTTCTGCCCCAAcctaccctacgtcctcctgc
**A15T top**	TTGGGGCAGAAAACTTGCAGAAACCGCACGTCCG
**A15T bottom**	CGGACGTGCGGTTTCTGCAAGTTTTCTGCCCCAAcctaccctacgtcctcctgc

**G17A top**	TTGGGGCAGAAAACTTACAGAAACCGCACGTCCG
**G17A bottom**	CGGACGTGCGGTTTCTGTAAGTTTTCTGCCCCAAcctaccctacgtcctcctgc
**P6_F3 top**	ATTAGGTTTATATAAGGTTTTAtacatcag
**P6_F3 bottom**	ctgatgtaTAAAACCTTATATAAACCTAATcctaccctacgtcctcctgc
**P8_F5 top**	TTTCTATGGGAGAAGTAAACCTAAACTATCTCCAAA
**P8_F5 bottom**	TTTGGAGATAGTTTAGGTTTACTTCTCCCATAGAAAcctaccctacgtcctcctgc
**P8_P9 top**	TTTCTATGGGAGAAGTAAAGGGGAAATGGACGCCCAA
**P8_P9 bottom**	TTGGGCGTCCATTTCCCCTTTACTTCTCCCATAGAAAcctaccctacgtcctcctgc
**F5_P9 top**	TTTTATGAAAGAAGGGGAAATGGACGCCCAAA
**F5_P9 bottom**	TTTGGGCGTCCATTTCCCCTTCTTTCATAAAAcctaccctacgtcctcctgc

Table S3. Crystallographic data collection and refinement statistics.

**Data collection**	
Space group	*H*3
Cell dimensions	
a, *c* (Å)	52.55, 54.88
Resolution (Å)	26.27–1.40 (1.44–1.40)
R_{merge}	0.019 (1.052)
< I / σI >	33.67 (1.42)
$CC_{1/2}$ (%)	100.0 (50.5)
Completeness (%)	100 (99.8)
Redundancy	5.1 (5.1)
No. unique reflections	11127
**Refinement**	
Resolution (Å)	26.27–1.40
No. reflections	11127
R_{work}/R_{free}	0.199/0.213
No. atoms	
Protein	445
Water	25
B-factors	
Protein	33.2
Water	42.0
R.m.s deviations	
Bond lengths (Å)	0.013
Bond angles (º)	1.6

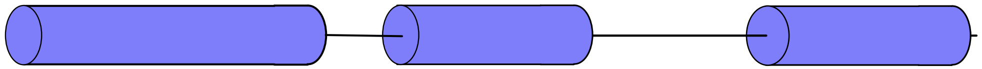

									PDB#	Q-score	%Identity
SPP1	8	TPKLERFVDE	YFINGM-NAT	KAAIAAGYSK	KSASTIAAEN	MQKP	50		-	-	79
SF6	7	SPKQERFIEE	YFINDM-NAT	KAAIAAGYSK	NSASAIGAEN	LQKP	49		4ZC3	-	-
hTRF1	385	WEEDKNLRSG	VRKYGEGNWS	KILLHYKFNN	-RTSVMLKDR	WRTM	427		1w0t	0.53	7
PrrA	143	RVRWEHIQRI	YEMCDR-NVS	ETARRLNM--	--HRRTLQRI	LAKR	181		1umq	0.5	18

Figure S1. Alignment of the amino acid sequences of α-helices 1-3 from HTH domains of SPP1 with SF6 small terminase (sequence alignment with clustal ω (1) showing 79% homology), and SF6 DBD with hTRF1 and PrrA (superposition with secondary structure matching, SSM PDBefold,(2)). Grey shading highlights residues that are conserved between the SF6 small terminase DBD and other HTH motifs, while the green shading denotes residues conserved between HTH motifs of hTRF1 and PrrA. SSM aligned α-helices are represented above as purple rods.

Table S4. PDBeFold secondary structure matching (SSM) statistics

	PDB ID	Q-score	Z-score	rmsd	N_{align}	N_{res}
**SF6**	4ZC3					
**hTRF1**	1W0T	0.53	5.1	1.87	41	52
**PrrA**	1UMQ	0.50	6.1	1.19	39	60

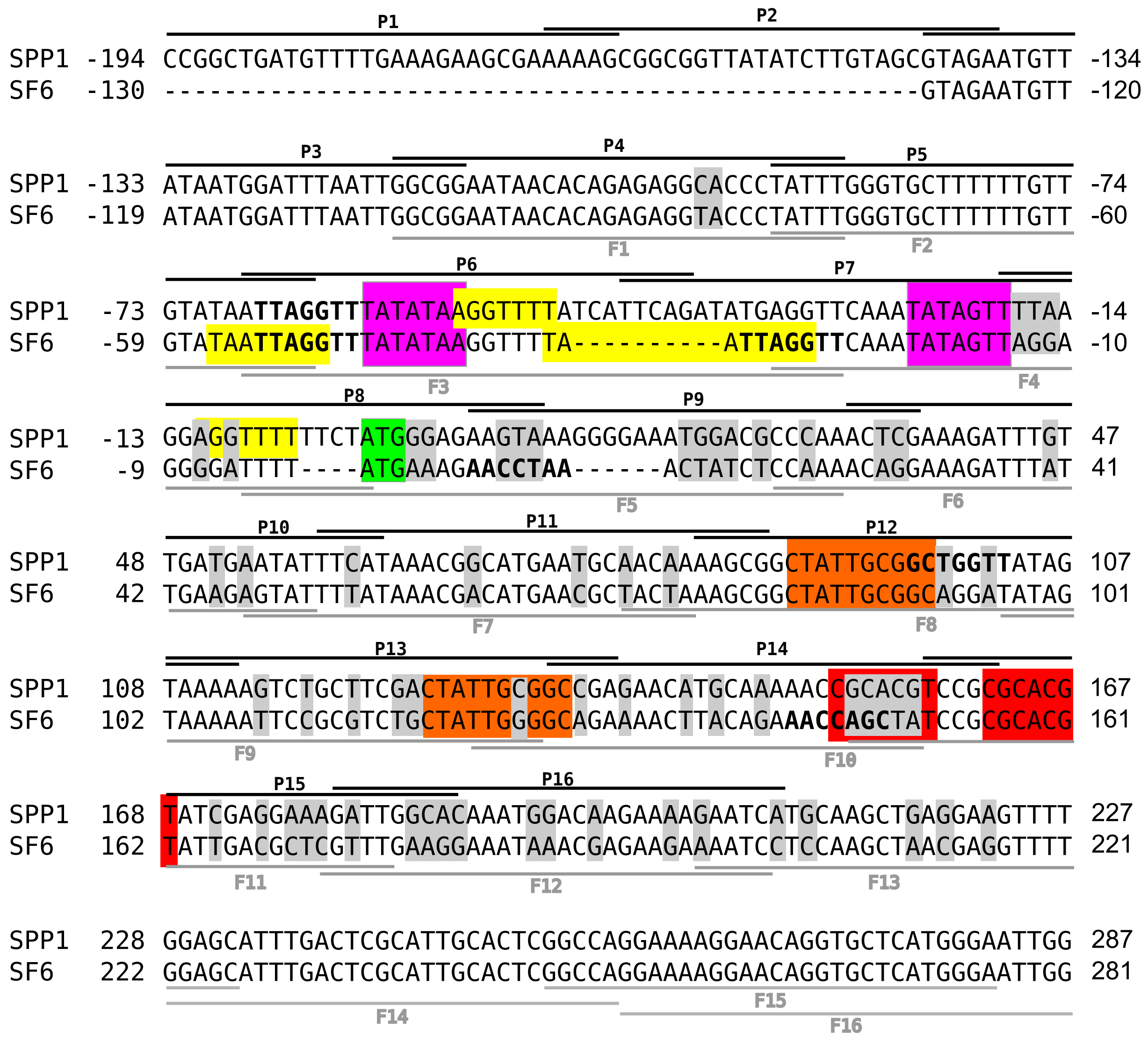

Figure S2. Alignment of *pac* site sequences from SPP1 and SF6 using clustal ω numbered relative to the start codon (bold and shaded in green). Grey shading indicates mismatches in the sequence. The purple boxes are the promoters PL1 and PL2, while the direct repeats of boxA (yellow shading), boxB (orange shading) and boxC (red shading) are noted. Oligonucleotide sequence segments used for ReDCaT screening (3) of DBD binding preference are labeled and represented by black and grey bars for SPP1 and SF6 pac sites respectively. Bold sequences are those identified as potential target motifs by MEME analysis.

Table S5. One-way ANOVA statistics for the ReDCaT screening assay.

**150 mM KCl**					
Source of VAR	SS	Df	MS	F	P
Between	0.4295	31	0.01386	1.277	0.18
Within	1.1169	103	0.01084		
Total	1.5464	134			
**300 mM KCl**					
Source of VAR	SS	Df	MS	F	P
Between	0.00416	31	0.000134	17.783	<0.05
Within	0.00078	103	7.546E-06		
Total	0.00494	134			
**Mutant sequences**					
Source of VAR	SS	Df	MS	F	P
Between	8.8229 E-07	17	5.190 E-08	17.9335	<0.05
Within	1.6785 E-07	58	2.894 E-09		
Total	1.0501 E-06	75			

ReDCaT analysis of SF6 DBB binding to pac sequences at 300 mM KCl concentration

Binding levels did not always correlate with the boxA, B or C motifs. For instance, while segments P6 and F3 (Figure 3A, Region 1) each contains a boxA site and bound greater amounts of DBD than the overall average, segments P8 and F2, which also contain boxA sites, did not (Figures 3A and S2, Table S2). Segments P12, P13 and F8, which contained boxB sites, or segments P14, P15 and F11, which contained boxC motifs, did not give rise to elevated binding. Conversely, segments F5 and F10 (Figure 3A, Regions 2 and 3, respectively) were not part of any sequence repeats and showed a greater-than-average binding density.

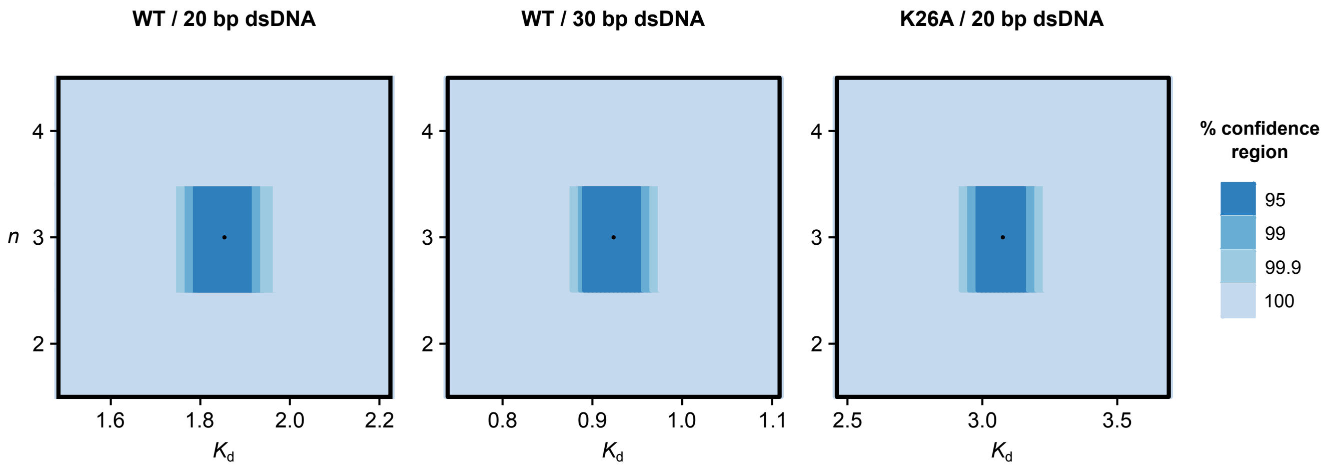

Figure S3. Residual sum of squares as a function of K_d and n under the Tsodikov-modified McGhee–von Hippel model for SPR data presented in Figures 5 and 7. Points indicate least-squares parameter estimates. Lines denote the 99% confidence regions as described by Beale (4) and shades (dark to light blue) correspond to the 95, 99, 99.9, 100 % confidence regions, respectively.

Analytical Ultracentrifugation.

Characterisation of the weak, rapidly reversible and non-specific association between the low molecular weight DBD domain and DNA is particularly challenging for several reasons, not least because of the weak binding that is dominated by non-specific interactions required a high concentration of reagents to detect protein-nucleic acid complex. To keep the amount of DBD required to form complex and reach saturation within a reasonable range, the minimum concentration of fluorescently labeled DNA required for detection by absorbance spectroscopy at the excitation wavelength was used (5 μM). Such low signal results in noisy data and fits that lead to residuals that are generally 10% or slightly more of the total signal. Further complications arise in the analysis of sedimentation velocity data for low molecular weight species such as DBD and, in particular, the hairpin and 11 basepair DNAs. Sedimentation rates of such species are often similar to the diffusion rates in the opposite direction, even at the maximum revolution rate of 60,000 rpm (5-7). As a result these small species often do not clear the meniscus efficiently and hence, the data is often difficult to analyse. To reduce this effect, the first 5 scans were removed from the data sets for the smaller species of DNA (11 basepair and hairpin DNA). Additionally, the different species of free DNA, free DBD and reversibly interacting DNA bound DBD all have very different hydrodynamic properties and shape which would be reflected in different values for frictional ratios, however, for simplicity and to avoid over-parameterization of this low signal data, the fits were all carried out using a frictional ratio that represented the average for all species. Finally, the high concentration of DBD required to form saturated complex resulted in changes in the overall density, viscosity and compressability of the solution (8-10). This is particularly evident in sedimentation profiles of the smaller DNAs (11 basepair and hairpin) in the highest concentration of DBD where the peak for the DBD bound DNA complex appears to be slightly smaller than for the species in the lower concentration of DBD. However despite all these caveats, the data for even the most complex or low signal experiments were fit reasonably well with a continuous c(s) distribution model giving rise to rmsd values less than 0.03 and residuals marginally more than 10% of the original signal, but generally random (see representative fit data of the two least quality fits, based on rmsd values, in Figure S4 and all rsmd values listed in Table S5; (7)).

A

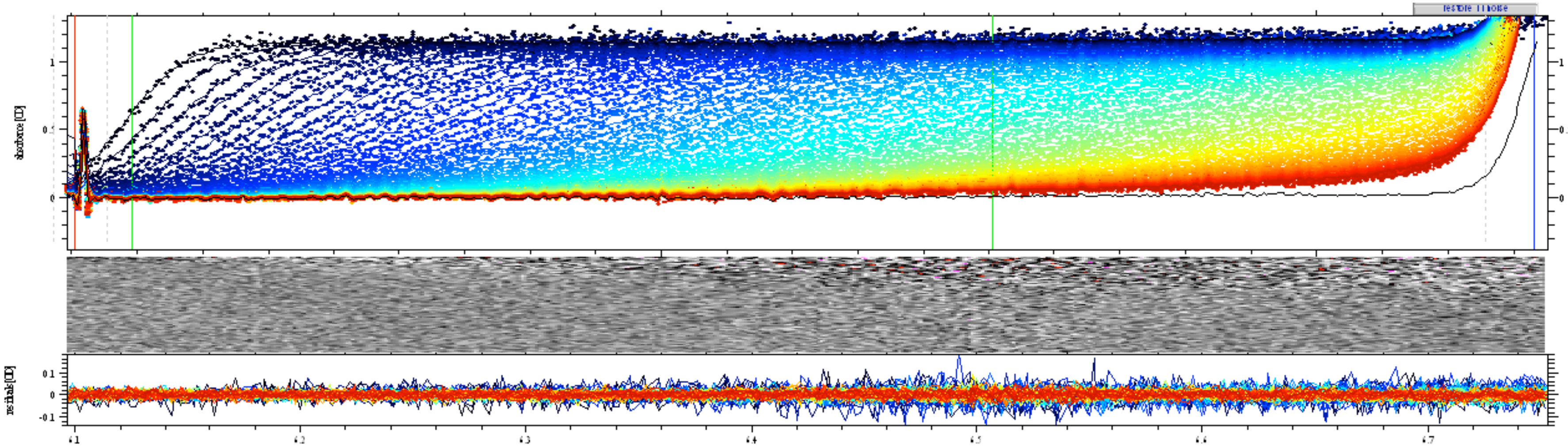

B

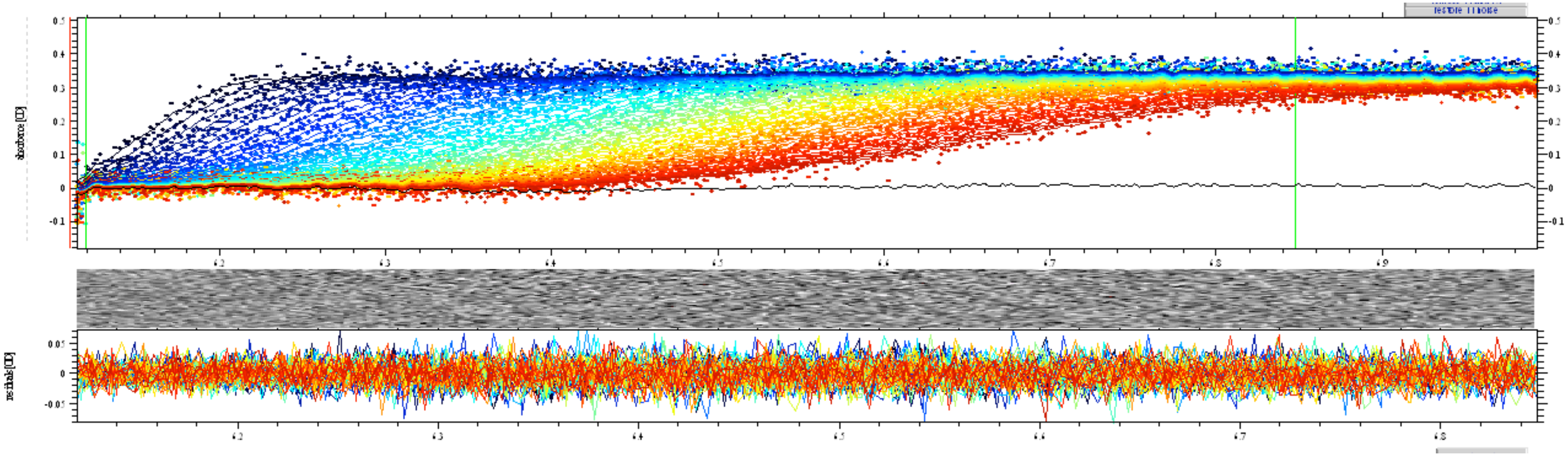

Figure S4. Sedimentation velocity absorbance data (datapoints, top panel) for the 30 basepair DNA (A) or 20 basepair DNA (B) fit to a continuous c(s) distribution model with Sedfit, The resulting fits to the data are show as lines in the top panel, the fit quality residuals as a bitmap (middle panel) and plotted as line graphs (lower panel)

Table S6. Fit parameters for each AUC experiment.

	rmsd	*f*/*f*$_0$	$s_{20,w}$ - %
**18C-hairpin**	0.0064	1.34	1.26 - 97
**+ ↓ [DBD]**	0.0064	1.359	1.27 - 55
**+ ↑ [DBD]**	0.0065	1.389	1.52 - 35
**14-nt hairpin + [DBD]**	0.0187	1.5	0.43 - 20<br>1.48 - 80
**11 bp DNA**	0.0182	1.348	1.76 – 97.5
**+ ↓ [DBD]**	0.0188	1.716	2.28 – 90.2
**+ ↑ [DBD]**	0.0189	1.559	2.17 – 85.6
**20 bp DNA**	0.0197	1.475	2.43 - 81
**+ ↓ [DBD]**	0.0187	1.662	2.96 – 67
**+ ↑ [DBD]**	0.0190	1.753	3.09 - 80
**30 bp DNA**	0.0226	1.662	2.22 – 21.8<br>2.9 - 67
**+ DBD**	0.0180	1.738	3.76 - 75

Conversion from c(s) profile to c(M) data and stoichiometry determination.

Since conversion from the c(s) profile is requires the correct value partial specific volume ($\bar{v}$) which differed with the number of DBDs bound to each DNA, the mass averaged $\bar{v}$ values were determined for each complex (with 1–6 DBDs bound to DNA, see Table S6 below) using an $\bar{v}$ of 0.74546 for DBD calculated using measured values at 25 °C (11-13) corrected to 20 °C (14) and an $\bar{v}$ value for DNA in 100 mM potassium chloride of 0.56 (6,15). These values were used to determine the molecular weight of each potential species and thus identify the stoichiometry of each protein bound DNA species (16).

Table S7. Partial specific volume ($\bar{\nu}$) calculations and observed molecular weight values for each protein-nucleic acid species.

	Theoretical MW (Da)	$\bar{\nu}$ per DBD in complex							Observed MW (kDa)
		0	1	2	3	4	5	6	
**DBD**	8251.6	N/A	0.7454	N/A	N/A	N/A	N/A	N/A	N/A
**18-C hairpin**	4223	0.56	0.68268	0.70767	0.71844	0.72443	0.72824	0.73089	4.34
**14-nt hairpin**	5084	0.56	0.67476						
**11 bp DNA**	7480	0.56	0.65728	0.68762	0.70243	0.71120	0.71700	0.72112	7.4
**20 bp DNA**	13430	0.56	0.63058	0.66225	0.68023	0.69182	0.69992	0.70589	13.47
**30 bp DNA**									
**(ss)**	10675	0.56							14.07
**(ds)**	20912	0.56	0.61248	0.64180	0.66053	0.67353	0.68308	0.69039	21.35

	$\bar{\nu}$ (for fit)	Theoretical MW per DBD in complex (Da)						Observed MW (kDa)	Proteins bound	Site size (bp)
		1	2	3	4	5	6			
**DBD**	0.7454	8251.6	16503.2	24754.7	33006.3	41257.9	49509.5			
**18-C hairpin + ↓ [DBD]**								4.34		
**18-C hairpin + ↑ [DBD]**	0.6826	12474.6	20726.2	28977.7	37229.3	45480.9	53732.5	11.07	1	
**18-C hairpin + ↑ [DBD] (repeat)**								12.9	1	≤5
**14-nt hairpin + [DBD]**	0.6747	13335.6						2<br>12.43	1	≤5
**11 bp + ↓ [DBD]**	0.6876	15731.6	23983.2	32234.7	40486.3	48737.9	56989.5	23.05	2	4–5
**11 bp + ↑ [DBD]**	0.7024	15731.6	23983.2	32234.7	40486.3	48737.9	56989.5	31.13	3	
**20 bp + ↓ [DBD]**	0.6802	21681.7	29933.3	38184.8	46436.4	54688	62939.6	39.04	3-4	4–5
**20 bp + ↑ [DBD]**	0.6918	21681.7	29933.3	38184.8	46436.4	54688	62939.6	47.9	4	
**30 bp + DBD**	0.6830	29163.6	37415.2	45666.7	53918.3	62169.9	70421.5	60.4	5	≤6

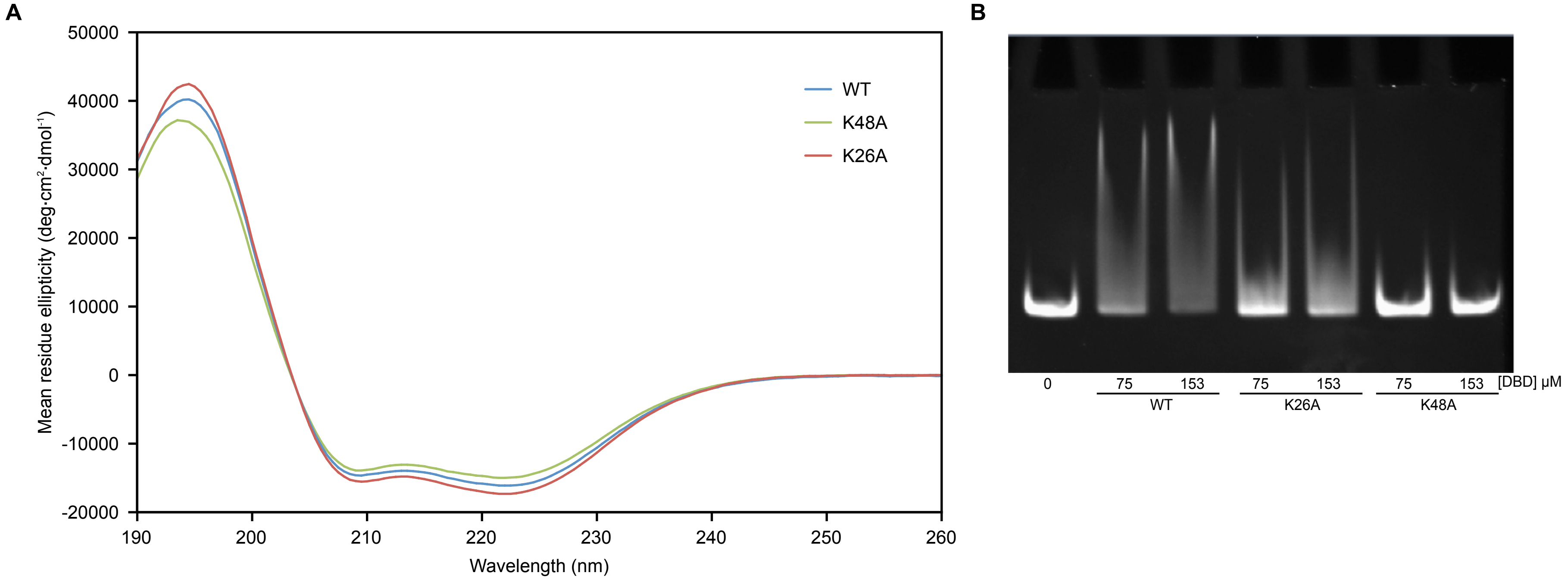

Figure S5. (A) Far-UV circular dichroism spectropolarimetry of wild-type (WT) DBD and mutants, K26A and K48A, at 0.2 mg/mL concentration in 10 mM Tris-Cl, pH 7.0, 25 mM Na_2SO_4. Spectra were recorded with 1-mm pathlength, 5 times, in a Jasco J810 CD spectropolarimeter, and averaged. (B) Electromobility shift assay with a 100-bp *pac* sequence DNA (1.5 μM; immediately upstream of the start codon, -100 to -1 bp in the SPP1 genome, Figure S3) with wild-type or mutant DBD. Protein and DNA were incubated for 1 h at 37 °C in 20 mM Tris-Cl, pH 7.5, 50 mM KCl, 10 mM $MgCl_2$, and subjected to electrophoresis at room temperature on a 7.5% polyacrylamide gel in 1× TAE. Gels were pre-run for 30 min prior to sample loading and DNA visualized with ethidium bromide.

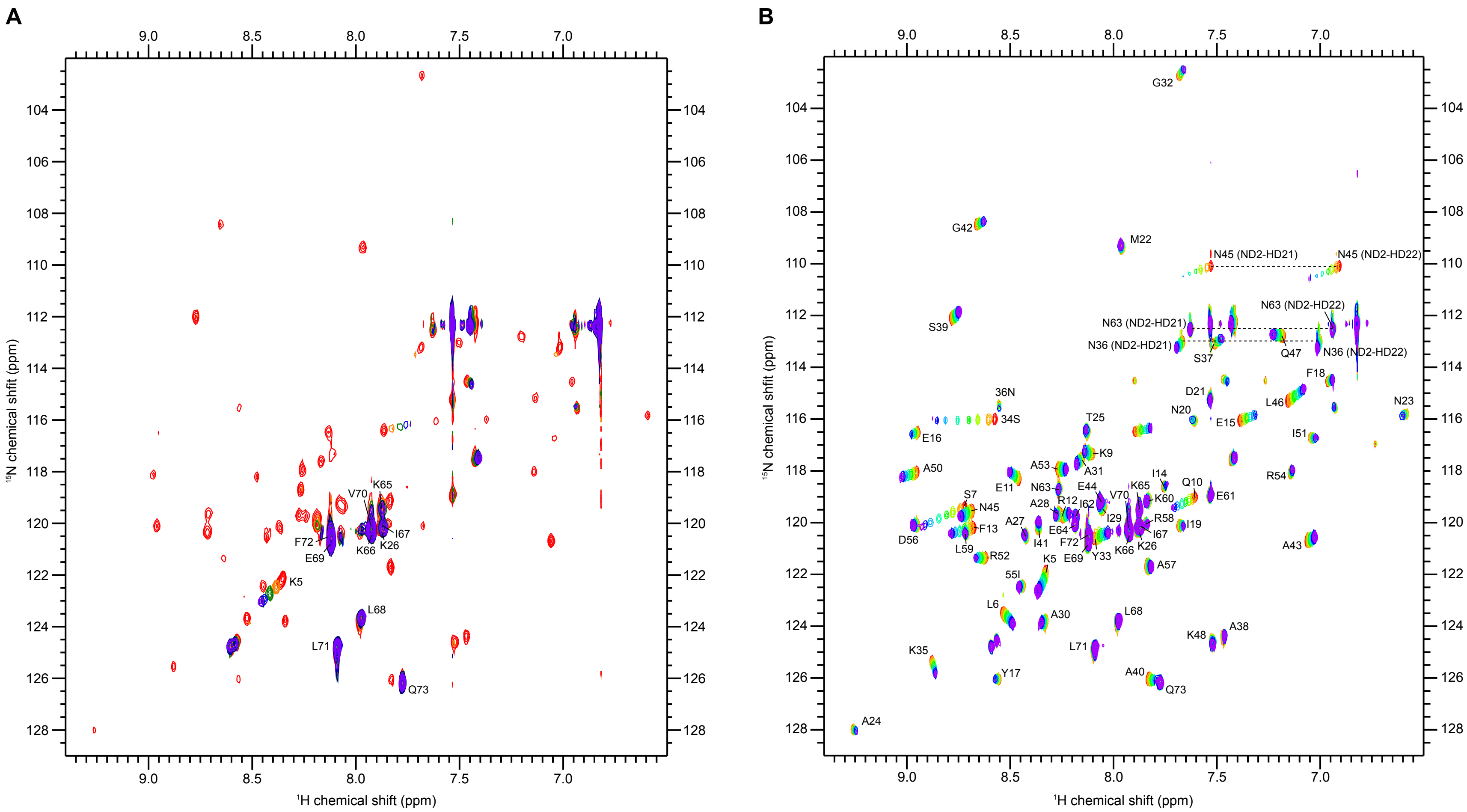

Figure S6. ^{1}H-^{15}N HSQC titration series (red to purple) of (A) 20-bp dsDNA and (B) 14-nt hairpin DNA into DBD at 25 °C in 20 mM MES, 150 mM KCl, pH 6.0, 10% D_2O, to final DNA/protein ratios to 1.5 and 19.2, respectively. Resonance assignments indicated.

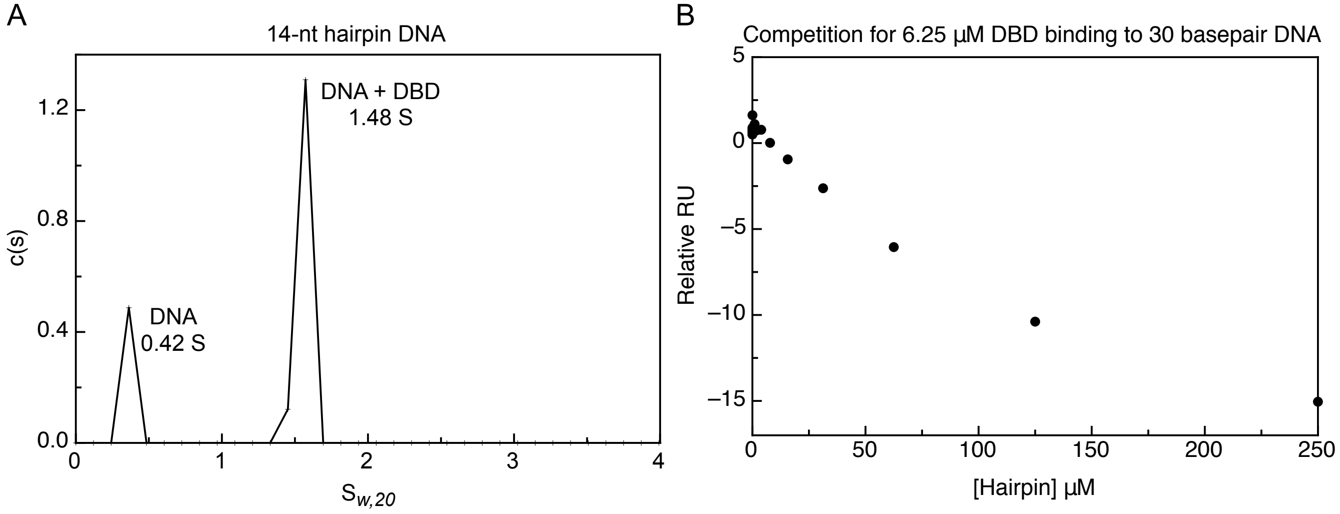

Figure S7. Analysis of DBD binding to a 5-bp hairpin DNA. (A). Sedimentation velocity AUC analysis of 6 μM 3′-ATTO 647N -labelled 14-nt hairpin DNA in complex with DBD (1.5 mM). Samples were run in 20 mM HEPES, 150 mM KCl, pH 7 at 60,000 rpm and 20 °C for 16 hours. Data were analysed as described with the caveats discussed above. (B) Competition SPR analysis with increasing concentrations of 18-C hairpin mixed with 6.25 μM DBD flowed over chip-immobilized 30 basepair DNA.

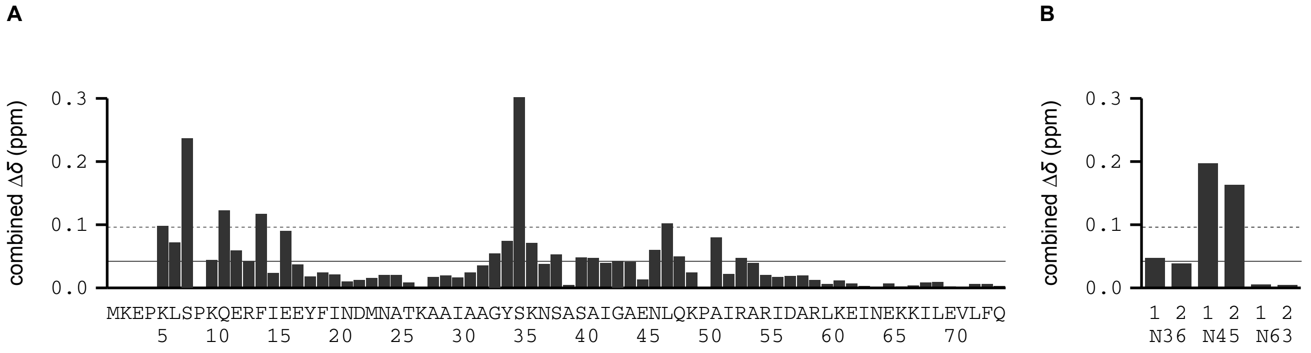

Figure S8. Combined chemical shift difference magnitudes of resonances in ^{1}H-^{15}N HSQC titration of 14-nt hairpin into DBD, calculated as detailed in Materials and Methods. Solid line indicates the mean combined magnitude, and dashed line indicates one standard deviation above mean.

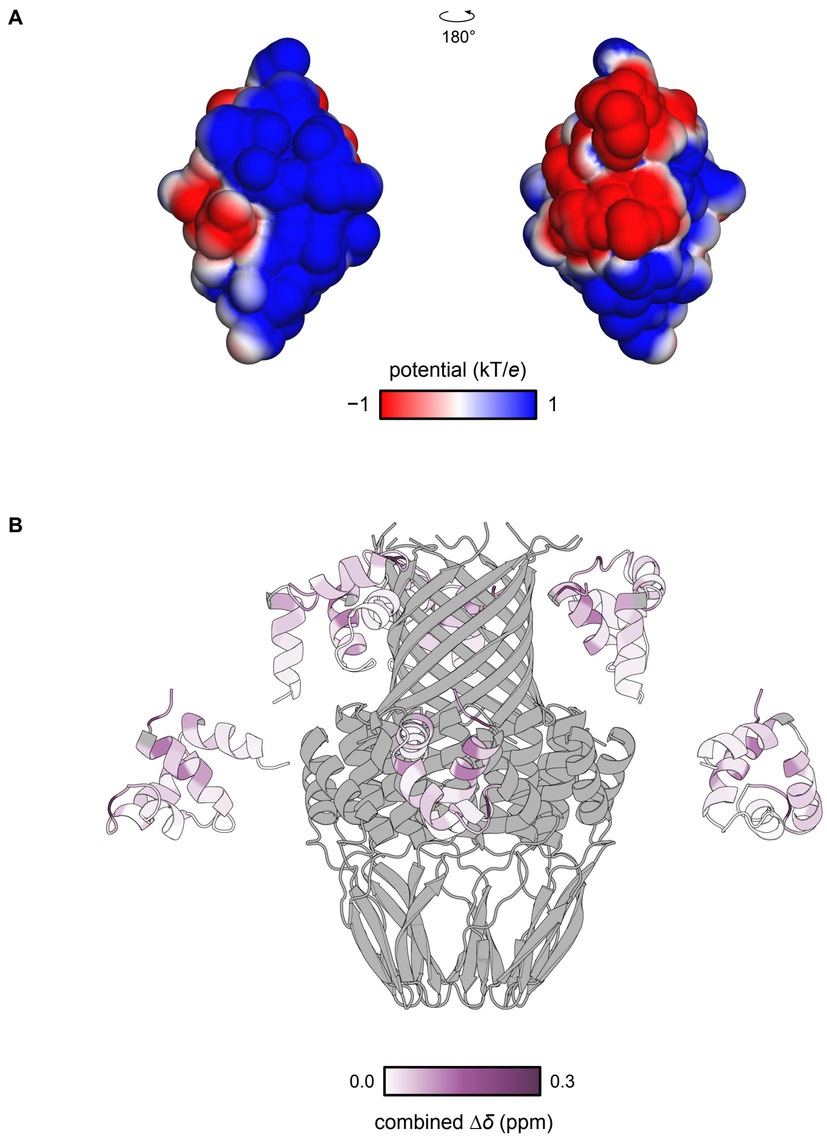

Figure S9. (A) Electrostatic potential of the solvent accessible surface of SF6 DBD under the SWANSON force field (17-20) in identical orientations as in Figure 7. (B) Combined chemical shift differences from ^{1}H-^{15}N HSQC titration of 14-nt hairpin DNA into DBD mapped onto the full-length oligomer structure (PDB ID 3ZQQ). Unassigned residues are coloured grey.

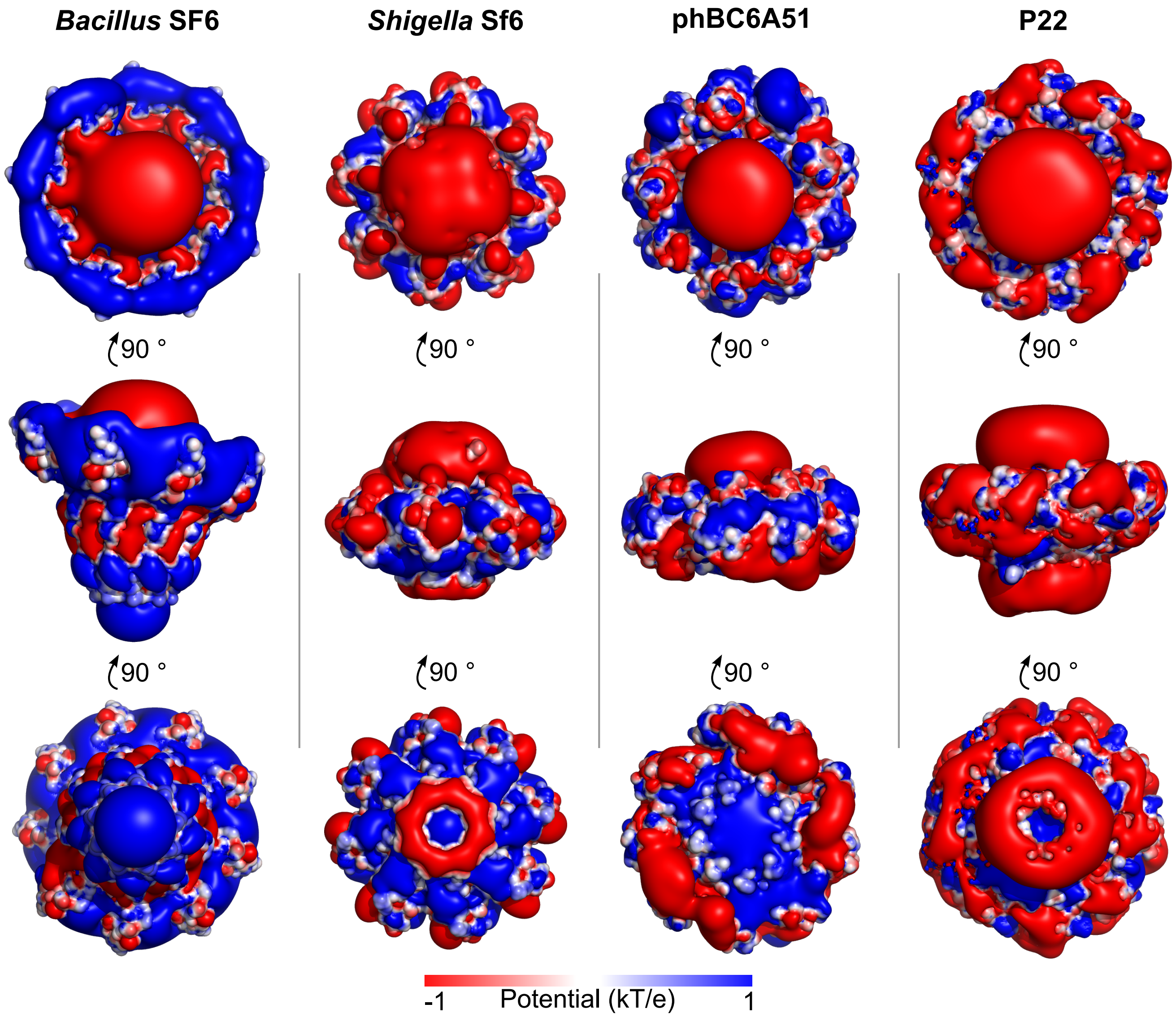

Figure S10. Electrostatic potential isocontours and potentials of the solvent-accessible surfaces of small terminase proteins (at 150 mM NaCl) under the SWANSON force field calculated using APBS (17-20)

References

1. Larkin, M.A., Blackshields, G., Brown, N.P., Chenna, R., McGettigan, P.A., McWilliam, H., Valentin, F., Wallace, I.M., Wilm, A., Lopez, R. *et al.* (2007) Clustal W and Clustal X version 2.0. *Bioinformatics*, **23**, 2947-2948.
2. Krissinel, E. and Henrick, K. (2004) Secondary-structure matching (SSM), a new tool for fast protein structure alignment in three dimensions. *Acta Crystallogr., Sect. D: Biol. Crystallogr.*, **60**, 2256–2268.
3. Stevenson, C.E., Assaad, A., Chandra, G., Le, T.B., Greive, S.J., Bibb, M.J. and Lawson, D.M. (2013) Investigation of DNA sequence recognition by a streptomycete MarR family transcriptional regulator through surface plasmon resonance and X-ray crystallography. *Nucleic Acids Res.*, **41**, 7009-7022.
4. Beale, E.M.L. (1960) Confidence regions in non-linear estimation. *J. R. Stat. Soc.: Series B (Stat. Methodol.)*, **22**, 41–88.
5. Schuck, P., MacPhee, C.E. and Howlett, G.J. (1998) Determination of sedimentation coefficients for small peptides. *Biophys. J.*, **74**, 466-474.
6. Hatters, D.M., Wilson, L., Atcliffe, B.W., Mulhern, T.D., Guzzo-Pernell, N. and Howlett, G.J. (2001) Sedimentation analysis of novel DNA structures formed by homo-oligonucleotides. *Biophys. J.*, **81**, 371-381.
7. Cole, J.L., Lary, J.W., T, P.M. and Laue, T.M. (2008) Analytical ultracentrifugation: sedimentation velocity and sedimentation equilibrium. *Methods Cell Biol.*, **84**, 143–179.
8. Schachman, H.K. (1959), *Ultracentrifugation in Biochemistry*. Academic Press, New York, pp. 174–180.
9. Millero, F.J., Ward, G.K. and Chetirkin, P. (1976) Partial specific volume, expansibility, compressibility, and heat capacity of aqueous lysozyme solutions. *J. Biol. Chem.*, **251**, 4001–4004.
10. Schuck, P. (2004) A model for sedimentation in inhomogeneous media. II. Compressibility of aqueous and organic solvents. *Biophys. Chem.*, **108**, 201–214.
11. Perkins, S.J. (1986) Protein volumes and hydration effects. The calculations of partial specific volumes, neutron scattering matchpoints and 280-nm absorption coefficients for proteins and glycoproteins from amino acid sequences. *Eur. J. Biochem.*, **157**, 169–180.
12. Laue, T., Shah, B., Ridgeway, T. and Pelletier, S. (1992) In Harding, S., Rowe, A. and Horton, J. (eds.), *Analytical Ultracentrifugation in Biochemistry and Polymer Science*. Royal Socity of Chemistry, UK, pp. 90–125.
13. Edelstein, S.J. and Schachman, H.K. (1967) The simultaneous determination of partial specific volumes and molecular weights with microgram quantities. *J. Biol. Chem.*, **242**, 306–311.

14. Durchschlag, H. (1986) In Hinz, H. (ed.), *Thermodynamic Data for Biochemistry and Biotechnology*. Springer Berlin Heidelberg, pp. 45–128.
15. Bonifacio, G.F., Brown, T., Conn, G.L. and Lane, A.N. (1997) Comparison of the electrophoretic and hydrodynamic properties of DNA and RNA oligonucleotide duplexes. *Biophys. J.*, **73**, 1532–1538.
16. Durchschlag, H. (1989) Determination of the partial specific volume of conjugated proteins. *Colloid Polym. Sci.*, **267**, 1139–1150.
17. Baker, N.A., Sept, D., Joseph, S., Holst, M.J. and McCammon, J.A. (2001) Electrostatics of nanosystems: application to microtubules and the ribosome. *Proc. Natl. Acad. Sci. U. S. A.*, **98**, 10037–10041.
18. Swanson, J.M.J., Wagoner, J.A., Baker, N.A. and McCammon, J.A. (2007) Optimizing the Poisson dielectric boundary with explicit solvent forces and energies: lessons learned with atom-centered dielectric functions. *J. Chem. Theory Comput.*, **3**, 170-183.
19. Dolinsky, T.J., Czodrowski, P., Li, H., Nielsen, J.E., Jensen, J.H., Klebe, G. and Baker, N.A. (2007) PDB2PQR: expanding and upgrading automated preparation of biomolecular structures for molecular simulations. *Nucleic Acids Res.*, **35**, W522-525.
20. Dolinsky, T.J., Nielsen, J.E., McCammon, J.A. and Baker, N.A. (2004) PDB2PQR: an automated pipeline for the setup of Poisson-Boltzmann electrostatics calculations. *Nucleic Acids Res.*, **32**, W665-667.
